# Phase I Study of Rogocekib in Patients with Advanced, Relapsed, or Refractory Malignant Solid Tumors

**DOI:** 10.1158/1078-0432.CCR-25-4896

**Published:** 2026-05-18

**Authors:** Jun Sato, Yuki Katsuya, Takafumi Koyama, Toshio Shimizu, Yasushi Tanoue, Maki Yamamoto, Hirokazu Tozaki, Eiji Takahara, Shingo Shoji, Akio Mizutani, Daisuke Morishita, Robert W. Oda, Hiroshi Miyake, Kan Yonemori, Noboru Yamamoto

**Affiliations:** 1 Department of Experimental Therapeutics, National Cancer Center Hospital, Tokyo, Japan.; 2 Department of New Experimental Therapeutics and Early-Phase 1 Drug Development Service, Kansai Medical University Hospital, Osaka, Japan.; 3 Chordia Therapeutics Inc., Fujisawa, Japan.; 4 Department of Medical Oncology, National Cancer Center Hospital, Tokyo, Japan.

## Abstract

**Purpose:**

Aberrant splicing plays a significant role in cancer progression, yet targeted treatments are lacking. Rogocekib (developmental name CTX-712) is a potent oral inhibitor of CDC2-like kinase that targets RNA splicing. This phase I study aimed to evaluate the maximum tolerated dose (MTD), dose-limiting toxicity (DLT), safety, tolerability, pharmacokinetics (PK)/pharmacodynamics (PD) profiles, preliminary efficacy, and the recommended dose of rogocekib in patients with advanced solid tumors.

**Patients and Methods:**

The dose escalation started with an accelerated titration phase and then transitioned to a 3 + 3 design at doses ranging from 10 to 175 mg, administered twice weekly. For dose expansion, 105 mg twice weekly, 70 mg twice weekly, and 105 mg once weekly were investigated.

**Results:**

In total, 46 patients with solid tumors were administered rogocekib. The MTD was determined to be 140 mg twice weekly. DLTs were observed in one patient at 140 mg twice weekly (platelet count decreased and hypokalemia) and in one patient at 175 mg twice weekly (dehydration). Common related adverse events included nausea, vomiting, and diarrhea. Two treatment-related deaths were observed. PK analysis showed a dose-dependent increase in systemic exposure to rogocekib. PD analysis of two markers (*THAP9-AS1* and *S6K*) showed target engagement of rogocekib. A partial response was observed in 6.5% of patients, all with ovarian cancer (*n* = 3).

**Conclusions:**

Although most toxicities were manageable, two treatment-related deaths were observed, underscoring the need for vigilant safety monitoring. Overall, rogocekib demonstrated evidence of target engagement in most patients with solid tumors and preliminary antitumor activity, warranting further clinical investigation.

**Translational Relevance**

Aberrant RNA splicing is a newly proposed hallmark of cancer progression and represents an underexplored therapeutic vulnerability. Rogocekib is a potent oral inhibitor of CDC2-like kinases (CLK), which regulate alternative splicing through phosphorylation of serine and arginine-rich proteins, critical components of the splicing machinery. By disrupting splicing, rogocekib induces cellular stress and promotes cancer cell death, particularly in tumors with *MYC* overexpression, a common feature in ovarian cancer and other solid tumors. This first-in-human phase I study demonstrated that CLK inhibition is clinically feasible, with evidence of target engagement and preliminary antitumor activity. The results from this study support the further development of rogocekib as a novel strategy for solid tumors, particularly in ovarian cancer and potentially in *MYC*-driven cancer.

## Introduction

RNA splicing is an essential mechanism for the proper expression of genes. The RNA machinery controls the inclusion of necessary coding fragments and excludes unnecessary stretches of pre-mRNA to create mature mRNA for protein expression. However, changes to the proper excision of pre-mRNA transcripts may lead to abnormalities within the cell. It has been reported that cancer cells exhibit a high level of alternative splicing (1, 2). These aberrant splicing patterns may contribute to cancer progression by transforming the tumor cells or by promoting resistance to anticancer therapies (3, 4). Mis-splicing can affect genes broadly across the transcriptome, but it can also occur during the transcription of specific tumor suppressor genes, contributing to cancer progression (5). In addition to these specific genes, mutations that occur in splicing factors that encode key regulatory proteins in the spliceosome (such as *SF3B1*, *SRSF2*, and *U2AF1*) have also been discovered, which are usually mutually exclusive and do not overlap with each other (6). As such, it may be possible to induce cell death in cancer cells by targeting the splicing machinery or splicing factors themselves.

There have been previous attempts to target splicing using small-molecule inhibitors or modulators that were evaluated in early phase I clinical studies. E7107 was a small-molecule inhibitor that targeted the SF3B spliceosome complex and disrupted spliceosome assembly by preventing tight binding of U2 small nuclear ribonucleoprotein to pre-mRNA (7). However, clinical evaluation of E7107 was discontinued due to severe vision-related toxicities and no efficacy signals above stable disease (SD; refs. 8, 9). In contrast, another splicing modulator, H3B-8800, acted on the SF3B complex and altered splicing through modulation of SF3B-dependent splice-site recognition (10). Although H3B-8800 was well tolerated and had no distinct safety concerns, the clinical efficacy was limited and did not progress past phase I (11). To date, there are still no approved medications that target splicing factors directly.

Rogocekib (developmental name CTX-712) is a potent, selective, and orally available small-molecule inhibitor of CDC2-like kinase (CLK) and presents a new mechanistically distinct approach to targeting splicing. The CLK family consists of four kinases, CLK1–4, and rogocekib can inhibit them all. CLKs are responsible for the phosphorylation of serine and arginine-rich (SR) proteins, which are critical components of the splicing machinery that interact directly with RNA and other proteins required for splicing. The inhibition of CLKs by rogocekib dephosphorylates SR proteins and can induce alternative splicing events (including exon skipping, intron retention, etc.), which can increase cellular stress. This increased cellular stress can lead to cell death in cancer cells already under elevated stress (12, 13). Preclinical studies of rogocekib revealed dose-dependent CLK inhibition, which led to SR protein dephosphorylation and widespread RNA mis-splicing events (14, 15). Among these altered splicing events, RNA markers *THAP9-AS1* and *S6K* showed dose-dependent changes preclinically and were selected to serve as clinical indicators of rogocekib-induced splicing modulation. Additionally, rogocekib has demonstrated antitumor activity in various *in vitro* and *in vivo* preclinical cell models, including splicing factor–mutated patient-derived xenograft models that show a positive correlation between splicing inhibition and increased antitumor efficacy (14). Furthermore, preclinical dose-finding experiments also showed that similar efficacy was seen when rogocekib was dosed in mice every day, twice weekly, and once a week while maintaining the same dose intensity throughout the week.

Preclinical results have also previously shown that CLK inhibition has a pronounced effect in cancers with the overexpression of the proto-oncogene *MYC. MYC* activation altered pre-mRNA splicing without the regulation of CLKs, which led to a synergistic vulnerability in cancer cells (16). *MYC* amplification is particularly prevalent in cancers such as ovarian cancer, in which 20% to 40% of all patients have *MYC* overexpression in their genetic profile (17, 18). Therefore, targeting the splicing machinery through CLK inhibition and exploiting vulnerabilities within the spliceosome in *MYC-*amplified tumors offers a potential novel strategy to treat patients with *MYC* overexpression.

To determine the safety profile of rogocekib, a phase I trial in malignant tumors was initiated in Japan. From toxicology studies of rogocekib in rats and dogs, the gastrointestinal (GI) tract was identified as the primary dose-limiting target organ, with additional effects in hematopoietic and lymphoid tissues and male reproductive organs. However, these findings were monitorable and largely reversible following dose interruption. After considering this safety information, taken together with the efficacy results seen in preclinical dose-finding experiments, twice-a-week dosing was chosen as the dosing regimen for clinical evaluation. Following an initial dose escalation cohort in solid tumors, additional expansion cohorts focusing on identifying candidate recommended dose (RD) levels were also initiated to confirm the safety profile of rogocekib. In this study, we report the final results from the phase I study of rogocekib in advanced, relapsed, and refractory solid tumors.

## Patients and Methods

### Study design

This was a first-in-human, open-label, phase I clinical study conducted in Japan in patients with advanced, relapsed, or refractory solid tumors and was registered on the Japan Registry of Clinical Trials under jRCT2080224127. The primary objectives of this trial were to assess the maximum tolerated dose (MTD) and the dose-limiting toxicities (DLT) to determine the RD for future clinical phases. The secondary objectives were to evaluate safety, tolerability, pharmacokinetics (PK), and pharmacodynamics (PD) and to preliminarily evaluate the antitumor efficacy of rogocekib. The exploratory objective was to explore the relationship between the clinical effect of rogocekib and biomarkers. The study was conducted in accordance with the Principles of the World Medical Association Declaration of Helsinki, all applicable Japanese Good Clinical Practices and regulations, and the Pharmaceutical and Medical Device Act for studies conducted in Japan and was approved by institutional review boards at participating sites. Written informed consent was provided by all patients before screening and enrollment.

### Patients

Patients ≥20 years of age with an Eastern Cooperative Oncology Group performance status of 0 or 1, with histologically or cytologically confirmed advanced or relapsed solid tumors, were considered eligible for the trial if they were unresponsive to standard therapies or were ineligible for appropriate standard therapies. Patients were enrolled in one of four cohorts (one dose escalation cohort A and three dose expansion cohorts C, D, and E; Supplementary Fig. S1). Cohort B was a dose escalation cohort for patients with hematologic malignancies; the results of this cohort were recently reported (19). As this current report focuses on patients with solid tumors, we have excluded a detailed investigation of cohort B from this article.

### Treatment

This phase I trial consisted of two parts: dose escalation and dose expansion. In the initial dose escalation cohort (A), the treatment initially followed an accelerated titration study design in which one patient was enrolled per dose level until a grade 2 or higher adverse event was observed. When this occurred, after consultation with the safety monitoring committee (SMC), an additional two patients were enrolled, and the study design then transitioned to a standard 3 + 3 design. Four dose levels were assessed in the accelerated titration phase (10, 20, 40, and 70 mg twice weekly), and three dose levels were assessed in the 3 + 3 phase (105, 140, and 175 mg twice weekly). Twice-weekly dosing was conducted on days 1 and 5 of every week of a 4-week cycle. After confirming tolerability, an additional 30 patients were enrolled in three dose expansion cohorts (C, D, and E). In cohorts C–E, rogocekib was administered orally at 105 mg twice weekly, 70 mg twice weekly, and 105 mg once a week (once weekly, dosed on day 1 of every week of a 4-week cycle), respectively.

### Safety assessments

The adverse events of rogocekib were assessed in accordance with the NCI Common Terminology Criteria for Adverse Events, version 5.0, translated into Japanese. Any toxicities that were considered to be related to rogocekib and that met the definition of a DLT were grade 4 or more thrombocytopenia persisting for more than 7 days or grade 3 or more thrombocytopenia accompanied by bleeding, grade 4 or more neutropenia persisting for more than 7 days, febrile neutropenia, grade 4 or more anemia, hematologic toxicities that required blood transfusion unrelated to underlying disease, grade 3 or more nonhematologic toxicities (except transient laboratory test abnormalities and diarrhea, nausea, vomiting, or other manageable systemic symptoms that resolved to grade 2 or less by appropriate treatments), and clinically significant grade 3 or more nonhematologic laboratory test abnormalities that did not resolve to grade 1 or less or to baseline within 7 days.

The DLT evaluation period was 28 days from the first dose of rogocekib (the duration of the first cycle) in the dose escalation part. A patient was considered DLT evaluable if they received 75% or more of the planned doses within the first cycle. Any patient who did not complete the DLT evaluation period for reasons other than DLT was not DLT evaluable and was replaced. However, if a patient had to discontinue treatment due to an adverse event, whether or not the adverse event constituted a DLT was determined by the SMC. DLTs were not assessed in cohorts C–E of the dose expansion part.

### PK/PD

For PK analyses in the dose escalation and for some patients in the expansion cohorts, peripheral blood samples were collected before and after the dose at 0.5, 1, 2, 4, 6, 8, and 24 hours on days 1 and 18, before the dose on days 4, 11, and 25 in cycle 1; before the dose on day 1 in cycle 2; and on day 1 of every even-numbered cycle thereafter. For other patients in the expansion cohort, peripheral blood samples were collected before and after the dose at 2 and 6 hours on day 1 of cycle 1, and before dose of day 1 in cycles 2 and 4. Plasma concentration of rogocekib was assayed with liquid chromatography–tandem mass spectrometry.

For PD analyses in the dose escalation and for some patients in the expansion cohorts, whole blood from patients was collected before and after the dose at 0.5, 1, 2, 4, 6, 8, and 24 hours on day 1 of cycle 1 using PAXgene Blood RNA Tubes (Becton, Dickinson and Company), and the RNA was isolated using the PAXgene Blood RNA Kit (QIAGEN N.V.). For other patients in the expansion cohort, peripheral blood samples were collected before and after the dose at 2 and 6 hours on day 1 of cycle 1, followed by RNA extraction. Following RNA isolation, the samples were assayed using the TaqMan RNA-to-C_T_*1-Step* Kit (Thermo Fisher Scientific Inc.) with samples run on the QuantStudio 5 (Thermo Fisher Scientific Inc.) system.

The inhibition of CLK induces genome-wide alternative splicing events (20). To track these changes, *THAP9-AS1* and *S6K* were chosen to confirm target engagement. A modified version of the 2^−ΔΔCt^ method was used to calculate the fold change (Fc) of the PD markers. The equation for Fc is given as follows:

$$\begin{array}{c} Fc=2^{-\Delta \Delta Ct} \end{array}$$ (A)

The calculation for ΔΔCt is defined as follows:

$$\begin{array}{c} \Delta \Delta Ct=\;\Delta Ct-\Delta {Ct}_{0} \end{array}$$ (B)

where ΔCt is defined as the ΔCt value at a specified time point (before or after the dose), and $\Delta {Ct}_{0}$ is the ΔCt value before the dose. The calculation for ΔCt is given by the following equation:

$$\begin{array}{c} \Delta Ct=x-y \end{array}$$ (C)

where x is defined as the threshold cycle number (Ct) of the skipping form of the target RNA and y is defined as Ct of the nonskipping form of the target RNA.

*GAPDH* was coamplified on the same plate to confirm the real-time PCR instrument’s function though not for normalization. Although running all samples on the same plate reduces noise, samples were processed as they arrived due to clinical trial logistics. Control and experimental samples for each patient were run together to ensure consistency and were run in batches when possible. If a Ct value was undetermined, it was assigned a value of 40 for calculations. The primer and probe sequences are listed in Supplementary Table S1.

### Biomarker analysis

Tumor tissues and cfDNA were analyzed for biomarker tests. Formalin-fixed paraffin-embedded tumor tissues were sequenced using the Oncomine Comprehensive Assay v3M (Thermo Fisher Scientific Inc.) on the Ion GeneStudio S5 Plus Sequencer (Thermo Fisher Scientific Inc.) or Ion S5 XL Sequencer (Thermo Fisher Scientific Inc.). The tumor tissues from patients 3 to 26 (20 samples) and patients 27 to 46 (18 samples) were sequenced by two different contract research organizations, so there were slight differences between the protocols of both organizations. Sequencing data of tumor tissues were analyzed using the Ion Reporter Software (Thermo Fisher Scientific Inc.). The detected short variants were filtered and selected based on the following criteria: being a nonsynonymous mutation or a mutation at the splice site, judged as PASS by the Ion Reporter Software, having a depth of ≥200, a variant frequency of ≥0.05, and no reports of an allele frequency ≥0.01 in either 1000 Genomes JPT Alt Allele Freq or HGVD All Alt Allele Freq. Furthermore, samples in which more than 100 C>T and G>A mutations were detected after these filters, C>T and G>A mutations with a variant frequency of ≤0.15 were excluded. In addition, in some patients within the expansion cohort, cfDNA samples collected around screening and, in some instances, around cycle 3 of day 1 and/or the end of treatment were sequenced using the TruSight Oncology 500 ctDNA v2 Kit (Illumina, Inc.) on the NovaSeq 6000 Sequencing System (Illumina, Inc.), followed by analysis using DRAGEN TruSight Oncology 500 ctDNA Analysis Software (Illumina, Inc.). In some of the above analyses, the predetermined quality in the protocol was not met. Based on the results of the above analyses, this article reports the analysis results of *MYC* CNV and short variants in *SF3B1* and *U2AF1* from tissue samples.

### Efficacy assessments

To assess the antitumor effect of rogocekib, tumor assessments were performed during screening, on day 1 of cycles 2 and 3, and on day 1 of subsequent odd-numbered cycles until disease progression was observed. The evaluation of the tumors was based on RECIST version 1.1. In cases where the best overall response was assessed as SD, it is required that (i) no progressive disease is observed from day 43 on or after the start of administration and (ii) at least one evaluation of SD or better [i.e., SD, partial response (PR), or complete response] is obtained on or after day 43. If the study treatment was terminated before disease progression was observed, tumor assessment was continued until confirmation of disease progression, the start of posttreatment, or death, depending on which occurred first. In all patients, response rate, tumor shrinkage rate, overall survival (OS), and progression-free survival (PFS) were analyzed as the efficacy endpoints. For survival analyses, long-term follow-up was performed every 6 months from the date of the last administration of rogocekib until the patient’s death, study completion, or 1 year from the start of rogocekib administration, whichever occurred first.

### Statistical analysis

The safety analyses included patients receiving at least one dose of rogocekib. Efficacy, PK, and PD analyses were performed on patients who received at least one dose of rogocekib and for whom relevant assessments were conducted or whose data were available. Descriptive statistics such as medians, standard deviations, and ranges were calculated. The Kaplan–Meier method was utilized for OS and PFS analysis. PK/PD parameters were assessed using a noncompartmental approach. Exploratory biomarkers were assessed through descriptive statistics.

## Results

### Patient characteristics

Among the 48 total patients enrolled, 46 patients received treatment, and all patients eventually discontinued treatment. Of the patients who received treatment, the median age was 56.0 years (range: 30–77 years), with 23.9% of patients being 65 years or older (11 patients in total). One hundred percent of the patients were Asian, representing a homogeneous patient population (Supplementary Table S2). One patient enrolled at 140 mg twice weekly in cohort A, and one patient in cohort E discontinued before the start of treatment. In the initial dose escalation part (cohort A), a total of 16 patients were administered rogocekib. One patient each was enrolled at 10, 20, 40, and 70 mg twice weekly. After the accelerated titration portion of the dose escalation, a 3 + 3 design was implemented, and 12 patients were treated at 105 mg (*n* = 3), 140 mg (*n* = 6), and 175 mg (*n* = 3) twice weekly. A majority of the patients in the dose escalation cohort were heavily pretreated with four or more previous lines of therapy (Table 1; Supplementary Table S3).

**Table 1.** Characteristics of patients in dose escalation and expansion cohorts.

Characteristic	Cohort A: 10 mg (*n* = 1), *n* (%)	Cohort A: 20 mg (*n* = 1), *n* (%)	Cohort A: 40 mg *(n* = 1), *n* (%)	Cohort A: 70 mg (*n* = 1), *n* (%)	Cohort A: 105 mg (*n* = 3), *n* (%)	Cohort A: 140 mg (*n* = 6), *n* (%)	Cohort A: 175 mg (*n* = 3), *n* (%)	Cohort C: 105 mg^a^ (*n* = 10), *n* (%)	Cohort D: 70 mg (*n* = 10), *n* (%)	Cohort E: 105 mg^b^ (*n* = 10), *n* (%)
Sex										
Male	0 (0)	1 (100)	0 (0)	1 (100)	2 (66.7)	5 (83.3)	2 (66.7)	4 (40)	2 (20)	2 (20)
Female	1 (100)	0 (0)	1 (100)	0 (0)	1 (33.3)	1 (16.7)	1 (33.3)	6 (60)	8 (80)	8 (80)
Age (years)										
Median	46	32	77	62	51	55	50	50.5	58	58.5
Range (min, max)	(46, 46)	(32, 32)	(77, 77)	(62, 62)	(51, 65)	(41, 68)	(45, 67)	(30, 68)	(32, 72)	(44, 72)
Age category (years)										
<65	1 (100)	1 (100)	0 (0)	1 (100)	2 (66.7)	5 (83.3)	2 (66.7)	9 (90)	6 (60)	8 (80)
≥65	0 (0)	0 (0)	1 (100)	0 (0)	1 (33.3)	1 (16.7)	1 (33.3)	1 (10)	4 (40)	2 (20)
Height (cm)										
Median	163	168	153	164	158	173.50	162	160	158	158.50
Range (min, max)	(163, 163)	(168, 168)	(153, 153)	(164, 164)	(155, 176)	(158, 184)	(157, 171)	(157, 168)	(155, 163)	(152, 163)
Weight (kg)										
Median	60.80	49.10	48.40	65	62.70	79.45	70	61.10	56.75	53.60
Range (min, max)	(60.8, 60.8)	(49.1, 49.1)	(48.4, 48.4)	(65, 65)	(53.1, 69.1)	(51.8, 99.7)	(61.8, 77.6)	(55.9, 66.7)	(52.1, 63.4)	(49.6, 60.6)
BMI (kg/m^2^)										
Median	22.88	17.40	20.68	24.17	22.31	25.75	25.07	22.80	22.46	22.30
Range (min, max)	(22.9, 22.9)	(17.4, 17.4)	(20.7, 20.7)	(24.2, 24.2)	(22.1, 25.1)	(18.4, 35.2)	(23.9, 29.6)	(19.8, 26.4)	(18.5, 25.7)	(20.2, 25.4)
Race										
Asian	1 (100)	1 (100)	1 (100)	1 (100)	3 (100)	6 (100)	3 (100)	10 (100)	10 (100)	10 (100)
Drinking history	​	​	​	​	​	​	​	​	​	​
Yes	1	1	0	1	2	6	3	7	8	7
No	0	0	1	0	1	0	0	3	2	3
Smoking history	​	​	​	​	​	​	​	​	​	​
Never smoked	1	0	1	0	2	2	1	4	5	8
Ex-smoker	0	1	0	1	1	4	2	4	5	2
Current smoker	0	0	0	0	0	0	0	2	0	0
Eastern Cooperative Oncology Group performance status at baseline										
0	1	1	1	1	1	6	3	8	8	6
1	0	0	0	0	2	0	0	2	2	4
2	0	0	0	0	0	0	0	0	0	0
≥3	0	0	0	0	0	0	0	0	0	0
Number of regimens										
0	0 (0)	0 (0)	0 (0)	0 (0)	0 (0)	0 (0)	0 (0)	0 (0)	0 (0)	0 (0)
1	0 (0)	0 (0)	0 (0)	0 (0)	0 (0)	0 (0)	0 (0)	0 (0)	0 (0)	0 (0)
2	0 (0)	0 (0)	0 (0)	0 (0)	0 (0)	1 (16.7)	0 (0)	1 (10)	1 (10)	0 (0)
3	0 (0)	0 (0)	0 (0)	0 (0)	1 (33.3)	0 (0)	0 (0)	2 (20)	1 (10)	1 (10)
≥4	1 (100)	1 (100)	1 (100)	1 (100)	2 (66.7)	5 (83.3)	3 (100)	7 (70)	8 (80)	9 (90)
Diagnosis										
Ovarian	0 (0)	0 (0)	1 (100)	0 (0)	1 (33.3)	1 (16.7)	1 (33.3)	3 (30)	4 (40)	3 (30)
Breast	1 (100)	0 (0)	0 (0)	0 (0)	0 (0)	0 (0)	0 (0)	0 (0)	1 (10)	2 (20)
NSCLC	0 (0)	0 (0)	0 (0)	0 (0)	0 (0)	0 (0)	1 (33.3)	0 (0)	2 (20)	2 (20)
Lung^c^	0 (0)	0 (0)	0 (0)	0 (0)	0 (0)	1 (16.7)	0 (0)	0 (0)	0 (0)	0 (0)
Mediastinal tumor	0 (0)	0 (0)	0 (0)	1 (100)	2 (66.7)	1 (16.7)	0 (0)	0 (0)	0 (0)	0 (0)
Colon	0 (0)	0 (0)	0 (0)	0 (0)	0 (0.0)	2 (33.3)	1 (33.3)	1 (10)	0 (0)	0 (0)
Renal cell carcinoma	0 (0)	1 (100)	0 (0)	0 (0)	0 (0)	1 (16.7)	0 (0)	0 (0)	0 (0)	0 (0)
Endometrial	0 (0)	0 (0)	0 (0)	0 (0)	0 (0)	0 (0)	0 (0)	0 (0)	2 (20)	1 (10)
Prostate	0 (0)	0 (0)	0 (0)	0 (0)	0 (0)	0 (0)	0 (0)	0 (0)	1 (10)	2 (20)
Pancreatic	0 (0)	0 (0)	0 (0)	0 (0)	0 (0)	0 (0)	0 (0)	1 (10)	0 (0)	0 (0)
Uterine	0 (0)	0 (0)	0 (0)	0 (0)	0 (0)	0 (0)	0 (0)	1 (10)	0 (0)	0 (0)
Bone and soft tissue tumor	0 (0)	0 (0)	0 (0)	0 (0)	0 (0)	0 (0)	0 (0)	1 (10)	0 (0)	0 (0)
Skin	0 (0)	0 (0)	0 (0)	0 (0)	0 (0)	0 (0)	0 (0)	2 (20)	0 (0)	0 (0)
Small intestine cancer	0 (0)	0 (0)	0 (0)	0 (0)	0 (0)	0 (0)	0 (0)	1 (10)	0 (0)	0 (0)

a 105 mg twice weekly.

b 105 mg once a week.

c Lung cancer that is not NSCLC.

### Safety

Across the dose escalation cohort (cohort A) and the dose expansion cohorts (cohorts C–E), treatment-related adverse events (TRAE) were common across dose levels and primarily grade 1 or 2 in severity. The most common TRAEs occurring in ≥30% of all patients with solid tumors (*n* = 46) were nausea (93.5%), vomiting (65.2%), diarrhea (54.3%), decreased appetite (45.7%), and blood creatinine increased (32.6%; Supplementary Table S4). Grade ≥3 TRAEs were reported in seven patients (15.2%) and are summarized in Table 2. In cohort A, no grade ≥3 TRAEs were observed at doses of 10, 20, 40, or 105 mg twice weekly. Grade ≥3 TRAEs were observed in one patient at 70 mg twice weekly (acute myeloid leukemia and myelodysplastic syndrome), one patient at 140 mg twice weekly (biliary tract infection, hypokalemia, hypophosphatemia, and platelet count decrease), and one patient at 175 mg twice weekly (dehydration, hypokalemia, and lymphocyte count decrease). However, no consistent safety pattern was observed across the dose levels. In the dose expansion part, grade ≥3 TRAEs were observed in one patient in cohort C (105 mg twice weekly; amylase increase, lipase increase, multiple organ dysfunction syndrome, esophageal ulcer, and platelet count decrease), one patient in cohort D (70 mg twice weekly; platelet count decrease), and two patients in cohort E (105 mg once weekly; diarrhea and hypotension). Consistent with cohort A, the TRAEs observed in the dose expansion phase were also heterogeneous and were not indicative of a consistent dose-dependent pattern.

**Table 2.** All grade ≥3 TRAEs occurring in all patients with solid tumors separated by cohort.

Preferred term	Cohort A: 10 mg (*n* = 1), *n* (%)^a^		Cohort A: 20 mg (*n* = 1), *n* (%)^a^		Cohort A: 40 mg (*n* = 1), *n* (%)^a^		Cohort A: 70 mg (*n* = 1), *n* (%)^a^		Cohort A: 105 mg (*n* = 3), *n* (%)^a^		Cohort A: 140 mg (*n* = 6), *n* (%)^a^		Cohort A: 175 mg (*n* = 3), *n* (%)^a^		Cohort C: 105 mg^b^ (*n* = 10), *n* (%)^a^		Cohort D: 70 mg (*n* = 10), *n* (%)^a^		Cohort E: 105 mg^c^ (*n* = 10), *n* (%)^a^	
Any event	0	(0)	0	(0)	0	(0)	1	(100)	0	(0)	1	(16.7)	1	(33.3)	1	(10)	1	(10)	2	(20)
Acute myeloid leukemia	0	(0)	0	(0)	0	(0)	1	(100)	0	(0)	0	(0)	0	(0)	0	(0)	0	(0)	0	(0)
Amylase increased	0	(0)	0	(0)	0	(0)	0	(0)	0	(0)	0	(0)	0	(0)	1	(10)	0	(0)	0	(0)
Biliary tract infection	0	(0)	0	(0)	0	(0)	0	(0)	0	(0)	1	(16.7)	0	(0)	0	(0)	0	(0)	0	(0)
Dehydration	0	(0)	0	(0)	0	(0)	0	(0)	0	(0)	0	(0)	1	(33.3)	0	(0)	0	(0)	0	(0)
Diarrhea	0	(0)	0	(0)	0	(0)	0	(0)	0	(0)	0	(0)	0	(0)	0	(0)	0	(0)	1	(10)
Hypokalemia	0	(0)	0	(0)	0	(0)	0	(0)	0	(0)	1	(16.7)	1	(33.3)	0	(0)	0	(0)	0	(0)
Hypophosphatemia	0	(0)	0	(0)	0	(0)	0	(0)	0	(0)	1	(16.7)	0	(0)	0	(0)	0	(0)	0	(0)
Hypotension	0	(0)	0	(0)	0	(0)	0	(0)	0	(0)	0	(0)	0	(0)	0	(0)	0	(0)	1	(10)
Lipase increased	0	(0)	0	(0)	0	(0)	0	(0)	0	(0)	0	(0)	0	(0)	1	(10)	0	(0)	0	(0)
Lymphocyte count decreased	0	(0)	0	(0)	0	(0)	0	(0)	0	(0)	0	(0)	1	(33.3)	0	(0)	0	(0)	0	(0)
Multiple organ dysfunction syndrome	0	(0)	0	(0)	0	(0)	0	(0)	0	(0)	0	(0)	0	(0)	1	(10)	0	(0)	0	(0)
Myelodysplastic syndrome	0	(0)	0	(0)	0	(0)	1	(100)	0	(0)	0	(0)	0	(0)	0	(0)	0	(0)	0	(0)
Esophageal ulcer	0	(0)	0	(0)	0	(0)	0	(0)	0	(0)	0	(0)	0	(0)	1	(10)	0	(0)	0	(0)
Platelet count decreased	0	(0)	0	(0)	0	(0)	0	(0)	0	(0)	1	(16.7)	0	(0)	1	(10)	1	(10)	0	(0)

a If a patient is reported to have the same event more than once, then the event with the most severe grade is counted once.

b 105 mg twice weekly.

c 105 mg once a week.

The TRAEs leading to discontinuation occurred in four patients (8.7%): one patient at 105 mg once weekly (grade 3 hypotension), one patient at 175 mg twice weekly (grade 3 dehydration), and the others were two patients with treatment-related deaths. TRAEs leading to dose reduction occurred in four patients (8.7%): one patient at 70 mg twice weekly (grade 2 diarrhea and nausea), one at 105 mg twice weekly (grade 1 nausea), one at 140 mg twice weekly (grade 3 platelet count decreased [without bleeding] and hypokalemia), and one at 175 mg twice weekly (grade 1 nausea). Antiemetics as concomitant medication were administered to 45 patients (97.8%). Out of 46 patients treated, four patients (8.7%) received treatment for more than 1 year, resulting in TRAEs in two patients (4.3%), all of which were grade 1 or 2, with no progressive worsening of adverse events detected over time.

The median total treatment duration for all solid tumor patients (*n* = 46) was 77 days (range: 1–596 days). During the dose escalation cohorts, three DLTs occurred in 2 of 16 patients (12.5%): hypokalemia and platelet count decreased (both grade 3) in one of six patients (16.7%) in the 140 mg twice weekly cohort and dehydration (grade 3) in one of three patients (33.3%) in the 175 mg twice weekly cohort.

The patient who experienced a DLT in the 175 mg twice weekly cohort presented with grade 2 nausea and vomiting as TRAEs shortly after the first dose and subsequently developed grade 2 diarrhea approximately 12 to 14 hours after the dose, which led to grade 3 dehydration accompanied by tachycardia, hypotension, and dyspnea. Laboratory evaluation revealed transient creatinine elevation and mild hepatic enzyme increases; additional urinary biomarkers (NAG and L-FABP) were normal, ruling out acute tubular injury. The event was attributed by the SMC to severe dehydration rather than direct drug toxicity. Given the severity of the DLT observed at 175 mg twice weekly and the interindividual variability in PK, the SMC recommended against enrolling additional patients at this dose level, which was allowed per the protocol.

Following the SMC recommendation to avoid further enrollment at 175 mg, three additional patients were enrolled at 140 mg twice weekly, resulting in a total of six patients. Among these, one patient experienced two treatment interruptions during the DLT evaluation period due to grade 3 thrombocytopenia and grade 3 hypokalemia. As per protocol, multiple interruptions were classified as a DLT. These laboratory abnormalities were transient and reversible after supportive care and dose reduction. No other DLTs occurred in the 140 mg twice weekly cohort. Consequently, the MTD was determined to be 140 mg twice weekly based on the SMC’s overall assessment of safety and PK data.

Dose expansion was initiated for further safety evaluation starting with cohort C, one dose level below the MTD of 140 mg twice weekly, in accordance with the SMC’s recommendation. This decision was based on the absence of DLTs at 105 mg twice weekly and the need to better characterize the safety and PK profile across a wider exposure range. DLTs were not evaluated in the dose expansion cohorts as per the protocol.

Treatment-related deaths were observed in two patients (4.3%): one patient at 140 mg twice weekly in cohort A (biliary tract infection) and one patient at 105 mg twice weekly in cohort C (esophageal ulcer and multiple organ dysfunction syndrome). The patient dosed with 140 mg twice weekly experienced septic shock triggered by grade 5 biliary tract infection, complicated by disseminated intravascular coagulation (DIC) and multiorgan failure. Imaging and laboratory findings indicated a severe biliary tract infection, including cholecystitis and suspected cholangitis, as the source of the infection. There was no evidence of drug-induced pneumonitis or immunosuppression. After a comprehensive review by the SMC, although there was no clear evidence that indicated that the study drug directly caused the event, the causal relationship with rogocekib could not ultimately be ruled out.

The patient dosed with 105 mg twice weekly developed an acute esophageal ulcer with bleeding during cycle 1 on day 5 following the second administration of the study drug, followed by shock, severe hypotension, and progressive multiorgan dysfunction despite intensive care. Endoscopy confirmed extensive esophageal ulceration requiring hemostasis. Laboratory findings showed grade 4 hepatic dysfunction, grade 3 renal dysfunction, and coagulation abnormalities consistent with DIC. Despite maximal supportive care, the patient died during cycle 1 on day 8. After extensive review, it was concluded that a causal relationship with rogocekib could not be denied, although the exact pathophysiology remained unclear.

### PK/PD

**Figure 1.**
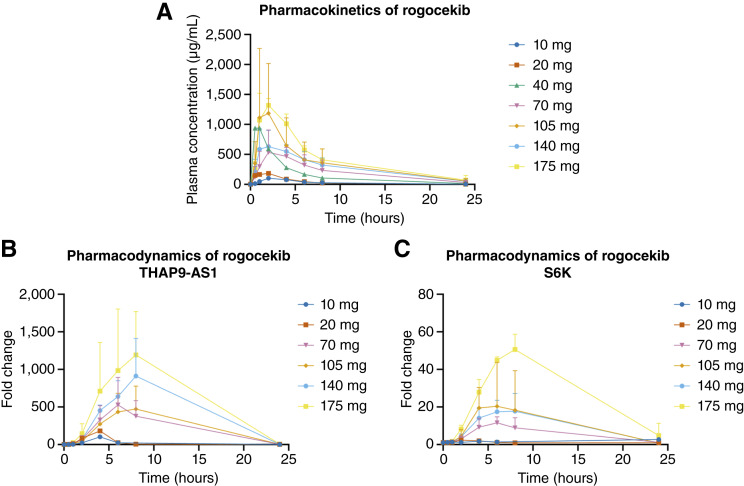
PK and PD of rogocekib. Mean plasma rogocekib concentrations following oral administration are shown (**A**). Error bars represent standard deviation (S.D.) values for each time point. Mean fold changes in THAP9-AS1 (**B**) and S6K (**C**) RNA markers following rogocekib administration are shown. Fold changes are presented as values relative to each patient’s predose baseline; accordingly, the predose value corresponds to a fold change of 1.0. Error bars represent S.D. values for each time point. For both PK and PD graphs, data from patients with a dose of 70 mg twice weekly from cohorts A and D have been combined for presentation purposes.

Rogocekib demonstrated a dose-dependent increase in systemic plasma concentration from samples collected in the dose escalation cohort (Fig. 1A). From plasma samples taken at cycle 1 on day 1, rogocekib was rapidly absorbed, with a median time to reach the maximum plasma concentration (T_max_) that ranged from 1 to 2 hours and was gradually eliminated, with a mean elimination half-life from time 0 to 24 hours after dose (T_1/2, 0–24 hours_) that ranged from 4.72 to 6.95 hours (Table 3). Both maximum plasma concentration (C_max_) and area under the plasma concentration–time curve from time 0 to 24 hours after dose (AUC_0–24_) at cycle 1 day 1 increased dose-dependently in a range from 10 to 175 mg. No accumulation was observed with repeated dosing. Across all dose levels, the relative dose intensity was >60% (Supplementary Table S5).

**Table 3.** PK parameters of patients with solid tumors.

Parameter	Cohort A	Cohort A	Cohort A	Cohort A	Cohort A	Cohort A	Cohort A	Cohort D
	10 mg twice weekly	20 mg twice weekly	40 mg twice weekly	70 mg twice weekly	105 mg twice weekly	140 mg twice weekly	175 mg twice weekly	70 mg twice weekly
*N*	*n* = 1	*n* = 1	*n* = 1	*n* = 1	*n* = 3	*n* = 6	*n* = 3	*n* = 5
C_max_ (ng/mL)	102 (–)	182 (–)	939 (–)	675 (–)	987.793 (125.5)	762.979 (23.6)	1,349.975 (12.2)	703.028 (27.2)
AUC_0–24 hours_ (hours·ng/mL)	599.604 (–)	876.940 (–)	3,692.515 (–)	2,929.903 (–)	6,034.485 (119.3)	5,823.618 (22.2)	9,048.775 (28.6)	4,724.754 (29.8)
T_max_ (hours)	2 (2, 2)	2.017 (2.02, 2.02)	1 (1, 1)	1 (1, 1)	1.983 (1, 2)	1.517 (1, 4)	1.933 (0.93, 1.98)	2.033 (1.95, 5.95)
T_½, 0–24 hours_ (hours)	4.722 (–)	4.809 (–)	5.364 (–)	4.150 (–)	6.945 (2.562)	5.887 (1.927)	5.626 (2.722)	5.873 (3.021)^a^

Values are summarized by cohort. C_max_ and AUC_0–24 hours_ are reported as geometric mean (%CV). T_max_ is reported as median (min, max). T_½, 0–24 hours_ is reported as arithmetic mean (SD).

a *n* = 4.

PD analyses were additionally performed on RNA isolated from peripheral blood samples. The relative magnitudes of exon skipping in target RNAs *THAP9-AS1* and *S6K* were quantified using real-time PCR (Fig. 1B and C). One patient who was administered 40 mg twice weekly was excluded from the PD analysis population due to the sample not being collected. The median time to reach the maximum Fc values (T_Emax_) in both *THAP9-AS1* and *S6K* ranged from 4 to 7.85 hours and 2.02 to 24.17 hours, respectively (Table 4). Like the PK profile, the maximum Fc values (E_max_) and area under the Fc values time curve from time 0 to 8 hours after dose (AUE_0–8 hours_) at cycle 1 day 1 in both *THAP9-AS1* and *S6K* increased dose-dependently in a range from 10 mg twice weekly to 175 mg twice weekly.

**Table 4.** PD parameters of patients with solid tumors.

​	Parameter	Cohort A	Cohort A	Cohort A	Cohort A	Cohort A	Cohort A	Cohort D
		10 mg twice weekly	20 mg twice weekly	70 mg twice weekly	105 mg twice weekly	140 mg twice weekly	175 mg twice weekly	70 mg twice weekly ^a^
	*N*	*n* = 1	*n* = 1	*n* = 1	*n* = 3	*n* = 6	*n* = 3	*n* = 5
THAP9-AS1	E_max_							
	Mean (S.D.)	102.8450 (–)	180.8940 (–)	268.4790 (–)	520.8903 (225.6245)	962.1078 (433.1844)	1,221.6527 (622.6646)	864.1922 (609.3825)
	Median (min, max)	102.8450 (102.845, 102.845)	180.8940 (180.894, 180.894)	268.4790 (268.479, 268.479)	554.8680 (280.204, 727.599)	942.9650 (427.861, 1,468.370)	974.6000 (760.427, 1,929.931)	593.0460 (397.460, 1,897.652)
	T_emax_ (hours)							
	Mean (S.D.)	4.083 (–)	4.000 (–)	5.833 (–)	6.617 (2.223)	6.583 (1.996)	7.094 (1.164)	7.403 (0.890)
	Median (min, max)	4.083 (4.08, 4.08)	4 (4, 4)	5.833 (5.83, 5.83)	7.850 (4.05, 7.95)	7.792 (3.97, 8)	7.767 (5.75, 7.77)	7.833 (5.82, 7.85)
	AUE							
	Mean (S.D.)	298.9329 (–)	525.1192 (–)	1,258.5746 (–)	1,958.0626 (891.0848)	3,228.0143 (924.3489)	4,924.0171 (3,748.6925)	2,733.9572 (1,669.9294)
	Median (min, max)	298.9329 (298.933, 298.933)	525.1192 (525.119, 525.119)	1,258.5746 (1,258.575, 1,258.575)	1,759.1238 [1,183.261, 2,931.803]	3,159.8107 [1,867.254, 4,467.766]	2,882.3050 [2,639.385, 9,250.362]	1,730.1596 [1,363.445, 5,298.780]
S6K	E_max_							
	Mean (S.D.)	2.6750 (–)	2.3440 (–)	15.3660 (–)	28.1610 (17.1201)	20.9538 (6.3074)	50.6563 (8.1384)	14.7410 (4.6480)
	Median (min, max)	2.6750 (2.675, 2.675)	2.3440 (2.344, 2.344)	15.3660 (15.3660, 15.3660)	25.1360 (12.755, 46.592)	20.3850 (11.956, 27.935)	49.6600 (43.062, 59.247)	13.4170 (11.223, 22.758)
	T_emax_ (hours)							
	Mean (S.D.)	24.167 (–)	2.017 (–)	4 (–)	5.322 (1.102)	6.286 (1.913)	7.800 (0.058)	5.883 (1.897)
	Median (min, max)	24.167 (24.17, 24.17)	2.017 (2.02, 2.02)	4 (4, 4)	5.950 (4.05, 5.97)	6.900 (3.97, 8)	7.767 (7.77, 7.87)	5.967 (3.95, 7.83)
	AUE							
	Mean (S.D.)	11.2555 (–)	11.9689 (–)	57.4656 (–)	105.8966 (83.0123)	88.7772 (22.4688)	214.0390 (15.4416)	59.6616 (12.0147)
	Median (min, max)	11.2555 (11.256, 11.256)	11.9689 (11.969, 11.969)	57.4656 (57.466, 57.466)	58.4559 (57.485, 201.749)	85.5474 (59.149, 119.286)	209.1862 (201.607, 231.324)	62.2730 (41.960, 74.316)

Abbreviation: S.D., standard deviation.

a Cohort D includes only subjects whose blood was collected eight times for PD evaluation of cycle 1 on day 1.

### Efficacy

**Figure 2.**
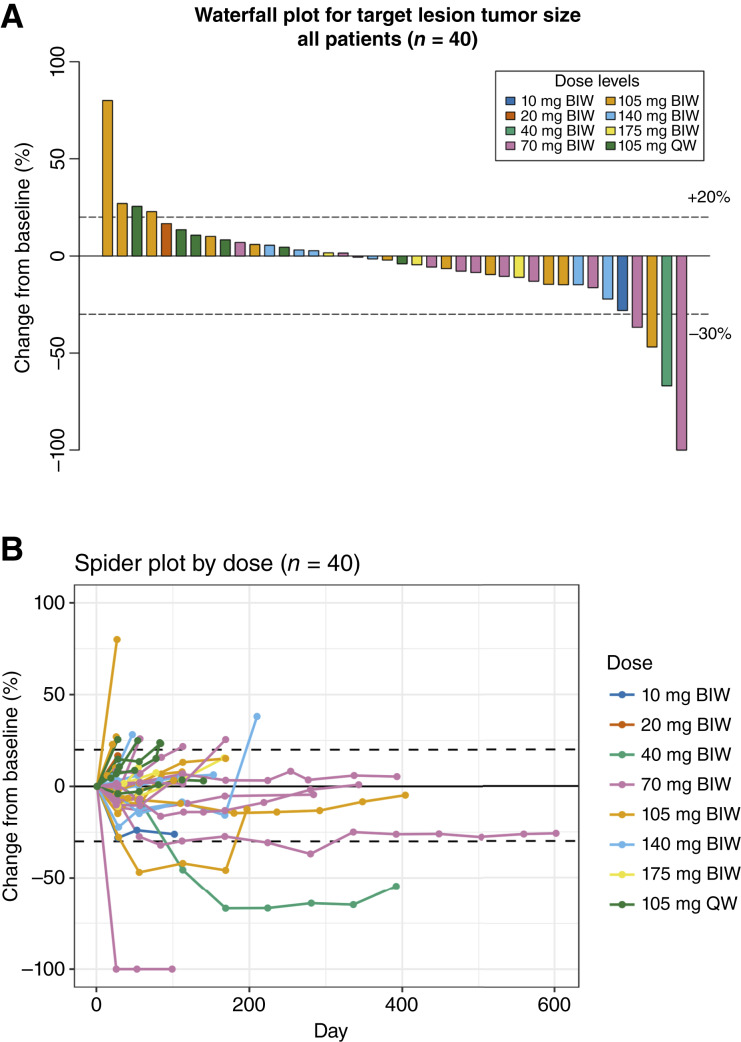
Efficacy of rogocekib in patients with solid tumors. Patients with measurable target tumor lesions were analyzed for maximum tumor shrinkage. The maximum percentages of tumor shrinkage are plotted in a waterfall plot (**A**). The dashed lines indicate RECIST thresholds for tumor reduction or growth. Spider plots of change in tumor size (%) over time were plotted for each dose level (**B**). Only tumors with target lesions were graphed on this spider plot. Dashed lines indicate RECIST thresholds for tumor growth or reduction.

Among all the patients in the solid tumor cohort (*n* = 46), PR was observed in three patients [all with ovarian cancer (high-grade serous carcinoma) at 40 mg twice weekly, 70 mg twice weekly, and 105 mg twice weekly], resulting in a response rate of 6.5% [95% confidence interval (CI), 1.4%–17.9%; Fig. 2A]. Two responses were observed in cohort A (40 mg twice weekly and 105 mg twice weekly), and one response was observed in cohort D (70 mg twice weekly). The patient who achieved PR in cohort D remained on study treatment for 596 days and was on treatment the longest among all patients in this study (Fig. 2B). One patient in cohort D had an observed tumor reduction of the main target lesion of 100%, but persistent nontarget lesions remained in the right supraclavicular, mediastinal, and para-aortic regions and were eventually determined to be progressive disease. According to the RECIST criteria, despite the total reduction of the main lesion, the best overall response was classified as SD. Of the three patients who had PR observed, two had detectable *MYC* gene amplification (Supplementary Figs. S2 and S3). The overall median OS and PFS were 9.61 months [95% CI, 6.28 months–not reached (NR); Supplementary Fig. S4A] and 3.68 months (95% CI, 1.87–5.49 months; Supplementary Fig. S4B), respectively.

## Discussion

This was a first-in-human phase I trial of the novel, first-in-class CLK inhibitor, rogocekib, which targets the spliceosome for patients with advanced, relapsed, or refractory solid tumors. Although previous attempts to target the splicing machinery have not demonstrated clinical efficacy (8), this study marks the first successful clinical advancement of a CLK inhibitor. This trial completed DLT evaluation, determined the MTD, and established candidate RD levels for future clinical evaluation. Overall, these results represent a significant step forward in the clinical development of therapies targeting RNA deregulation stress in cancer.

Rogocekib was generally associated with a manageable safety profile throughout the study. The most common adverse events of this trial were GI-related toxicities such as vomiting, nausea, and diarrhea, and they are consistent with other phase I trials assessing splicing modulators such as H3B-8800 and E7107 in solid tumors, which also reported GI toxicities as the main adverse events (8, 9, 21). These GI-related adverse events were manageable and brought to a tolerable state with the administration of concomitant medication including antiemetics. These toxicities are consistent with the safety profile observed from the administration of rogocekib in patients with hematologic malignancies and were also managed with the concomitant use of antiemetics (19). For patients administered rogocekib for over a year, TRAEs were minimal and mostly low grade, suggesting a favorable safety profile in long-term administration. In addition, there was no evidence of drug-induced QTc prolongation for rogocekib, which is also consistent with the safety profile of other splicing modulators (9, 11).

The DLTs were observed at 140 mg twice weekly (platelet count decreased and hypokalemia) and at 175 mg twice weekly (dehydration). The DLT observed at 175 mg twice weekly, in particular, raised concerns due to its relatively rapid onset (only one dose without any readministration) and clinical severity, which included grade 3 dehydration accompanied by tachycardia, hypotension, and dyspnea. The SMC reviewed this case extensively, noting that the severity of the diarrhea, combined with interindividual PK variability, posed a safety risk if additional patients were enrolled at 175 mg twice weekly. Based on these findings, the SMC recommended enrolling three additional patients to confirm safety at 140 mg twice weekly instead of 175 mg twice weekly. This decision deviated from the conventional 3 + 3 design; however, this was permitted by the study protocol when a recommendation was made by the SMC to mitigate the risk of excessive exposure and prioritize patient safety.

After the enrollment of an additional three patients at 140 mg twice weekly, DLTs (platelet count decreased and hypokalemia) were observed in one patient, which meant one DLT was observed out of a total of six patients. Importantly, the DLT in the patient dosed at 140 mg twice weekly was driven by protocol-defined criteria (two interruptions during the DLT evaluation period) rather than irreversible toxicity. As such, 140 mg twice weekly was determined to be the MTD.

Based on the recommendation from the SMC, dose expansion was initiated at 105 mg twice weekly, a dose that had no observed DLTs at the time of expansion. Safety and preliminary efficacy were subsequently evaluated at 70 mg twice weekly, 105 mg twice weekly, and 105 mg once weekly; however, DLTs were not assessed during the expansion phase.

Altogether, two TRAEs resulted in deaths in patients dosed at 140 mg twice weekly in the dose escalation cohort and at 105 mg twice weekly in the dose expansion cohorts. A causal relationship with rogocekib could not be ruled out, and these events were considered serious safety findings requiring careful evaluation. However, detailed evaluation suggested that neither event represented a clear or consistent safety signal for rogocekib.

The cause of death in the patient dosed at 140 mg twice weekly in the dose escalation cohort was most likely due to severe biliary tract infection (cholecystitis/cholangitis) rather than drug-related toxicity. The patient’s death was preceded by a severe fever around 40°C for a few days, followed by multiorgan failure despite intensive supportive care. Imaging revealed gallbladder swelling, ascites, and mesenteric fat stranding, consistent with cholecystitis and cholangitis, and pneumobilia was present at baseline, indicating preexisting biliary vulnerability. Emergency evaluation revealed septic shock and multiorgan failure, with imaging findings consistent with acute biliary tract infection. There was no documented history of repeated or chronic biliary tract infection prior to study entry. After full review, the cause of death was determined to be sepsis secondary to acute biliary infection, and a causal relationship with rogocekib could not be excluded due to the absence of an autopsy and the unclear underlying pathology.

The patient dosed at 105 mg twice weekly in the dose expansion cohorts experienced acute esophageal ulceration with bleeding, followed by shock and multiorgan failure. Despite extensive evaluation, including endoscopy, imaging, infectious disease testing, and PK analysis, the underlying pathophysiology could not be fully explained. Although bleeding from the ulcer contributed to hemodynamic instability, the shock-like state and systemic edema were disproportionate to the ulcer alone. Possible mechanisms such as drug-induced allergy or capillary leak syndrome were considered but not confirmed. Given the temporal association with rogocekib administration and the lack of alternative explanations, a causal relationship could not be ruled out. However, no other patient in the trial experienced ulcers as an adverse event due to dosing with rogocekib. Similarly, there were no reports of GI bleeding or ulcers observed in other splicing modulators (9, 11). Overall, these events highlight the importance of close monitoring for GI complications and infection risk in patients receiving rogocekib. However, both cases seemed to involve complex, multifactorial processes rather than a consistent drug-related toxicity pattern, and no similar events were observed in other participants.

Neither fatal event met the protocol definition of a DLT; one death occurred on day 46 of treatment in the dose escalation cohort, which was outside the predefined DLT evaluation period, and the other occurred in the dose expansion cohort, during which DLTs were not formally assessed. Nevertheless, both events were reviewed in detail by the SMC as treatment-related deaths during the study period, with careful consideration of the potential causal relationship with rogocekib. However, no specific biliary or esophageal target-organ toxicities, nor evidence of a distinct off-target signal, were identified in preclinical safety pharmacology or repeat-dose toxicology studies. Altogether, although both cases seemed to involve complex, multifactorial processes rather than a consistent or dose-dependent toxicity pattern, targeted safety measures were implemented following SMC review, and no similar events were observed in other participants.

PK analysis of patients who received rogocekib showed a systemic increase in plasma concentration in a dose-dependent manner. The fold change in the relative amounts of exon skipping of the PD markers *S6K* and *THAP9-AS1* also increased in a dose-dependent manner and was concordant with the increase in the concentration of rogocekib. These results confirm the target engagement of rogocekib and its ability to inhibit CLKs and induce splicing changes in humans.

Preliminary efficacy data showed that three patients who received rogocekib achieved PR, all of whom had ovarian cancer (high-grade serous carcinoma in all three; Supplementary Fig. S5). Coincidentally, two of these patients also had *MYC* gene amplification (Supplementary Table S6). Compared with the overall response rate (ORR) across all patients with solid tumors (6.5%), the ORR for patients with ovarian cancer was higher (21.4%), which suggests a potential sensitivity in ovarian cancer to rogocekib (Supplementary Table S7). Additionally, patients with ovarian cancer tended to have better OS and PFS, especially those with *MYC* amplification (Supplementary Fig. S6). However, due to the exploratory nature of efficacy in this phase I study, efficacy comparisons across dose levels should be interpreted with caution. Specifically, during the dose escalation phase, responses were seen at 40 mg twice weekly and 105 mg twice weekly, but these dose levels included very small numbers of patients (*n* = 4 total across these doses); additionally, another response was observed at 70 mg twice weekly in cohort D of the expansion cohort. However, the overall number of patients per cohort was limited; together with the variability in OS and PFS (Supplementary Fig. S7), any definitive conclusion about efficacy or dose dependency is difficult. As such, larger cohorts will be required to reliably assess antitumor activity and exposure–response relationships for rogocekib.

Altogether, the results from this trial suggest that rogocekib has a tolerable safety profile and has demonstrated preliminary efficacy in solid tumors, especially in ovarian cancer. Although 140 mg twice weekly was determined by the SMC to be the MTD, the number of patients treated at each dose level in the expansion cohorts was limited, and there was insufficient evidence to definitively identify an RD. All three regimens evaluated in the expansion cohorts, 70 mg twice weekly, 105 mg twice weekly, and 105 mg once weekly, were therefore considered to be candidate RD levels, but a definitive RD was not determined.

To establish the RD, further accumulation of patients at each dose level, more detailed evaluation of PK/PD and efficacy dose–response relationships, and comparison of optimal dosing schedules (e.g., twice weekly vs. once weekly), particularly around the 105 mg dose level, will be required. These aspects could not be fully addressed within the scope of this phase I study. Therefore, in line with the principles of Project Optimus, this study was designed to inform dose optimization rather than establish a single definitive RD, and the final determination of the RD will be explored in subsequent clinical studies with an emphasis on balancing safety and efficacy. Currently, rogocekib is under active clinical investigation in the United States in a phase I/II trial in patients with relapsed or refractory acute myeloid leukemia or myelodysplastic syndromes, evaluating rogocekib in a new tablet formulation at doses ranging from 20 to 140 mg once weekly or 60 to 100 mg twice weekly (NCT05732103).

Overall, although the safety profile was mostly tolerable, the emergence of treatment-related deaths underscores the need for vigilant and proactive safety monitoring. However, the early evidence of target engagement and the preliminary efficacy signals from this phase I trial demonstrate that there is potential within this class of medication and serve as an indication that there is a mechanistic rationale for the treatment of cancer with CLK inhibition. Collectively, these findings support the continued clinical development of rogocekib and further evaluation of CLK inhibition as a therapeutic strategy in cancer.

## Supplementary Material

Figure S1 Study design of CTX-712-Cl-01.

Figure S2 Waterfall plot of maximum tumor shrinkage in patients evaluated at doses in dose expansion.

Figure S3 Waterfall plot of maximum tumor shrinkage in patients with ovarian cancer.

Figure S4 OS and PFS of all patients with solid tumors.

Figure S5 Spider plot of changes in tumor size for all patients with ovarian cancer.

Figure S6 OS and PFS of patients with ovarian cancer.

Figure S7 OS and PFS for patients evaluated at doses in dose expansion.

Table S1 Primer and probe sequences for PD analysis.

Table S2 Representativeness of Study Participants.

Table S3 Expanded previous therapies for patients with solid tumors.

Table S4 All TRAEs occurring in ≥10% of patients with solid tumors.

Table S5 Dose intensity per dose level in patients with solid tumors.

Table S6 Biomarker analysis of tissue samples from patients with solid tumors.

Table S7 ORR of all patients with solid tumors and ovarian cancer.

## Data Availability

Chordia Therapeutics supports responsible data transparency to advance scientific understanding while safeguarding participant confidentiality. Anonymized data may be made available to qualified researchers upon request, following completion of all planned analyses and regulatory approval of the indication. Requests will be reviewed for scientific merit and feasibility, and access will be granted upon execution of a data use agreement. For inquiries, please contact us at https://www.chordiatherapeutics.com/en/contact.html.
