# Supplementary material for: Phase I Study of Rogocekib in Patients with Advanced, Relapsed, or Refractory Malignant Solid Tumors
Source: Clin Cancer Res. 2026 May 18;32(15):3115–25. doi: 10.1158/1078-0432.CCR-25-4896 (PMC13430218; doi:10.1158/1078-0432.CCR-25-4896)
Supplement: Figure S1 — Study design of CTX-712-Cl-01. [file ccr-25-4896_figure_s1_suppfs1.docx]

Figure S1


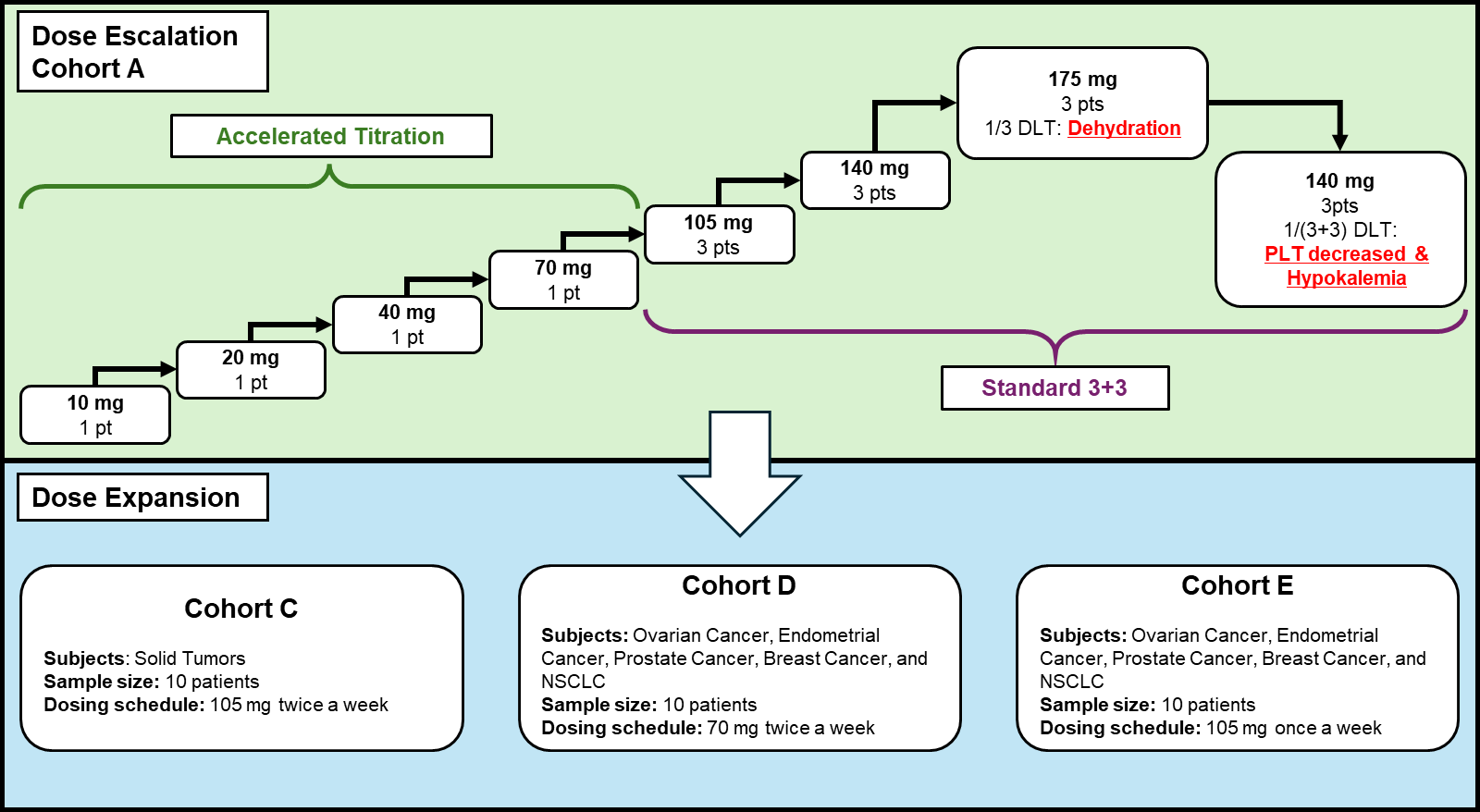


**Figure S1: Study design of CTX-712-Cl-01.**

This phase 1 study included dose escalation (cohort A) and dose expansion (cohorts C–E). Dose escalation began with an accelerated titration design (10–70 mg BIW) and transitioned to a 3+3 design at 105–175 mg BIW. BIW dosing was administered on Days 1 and 5 of each 4‑week cycle. Following confirmation of tolerability, dose expansion evaluated rogocekib at 105 mg BIW (cohort C), 70 mg BIW (cohort D), and 105 mg QW (Day 1 of each 4‑week cycle; cohort E).
