# Supplementary material for: Phase I Study of Rogocekib in Patients with Advanced, Relapsed, or Refractory Malignant Solid Tumors
Source: Clin Cancer Res. 2026 May 18;32(15):3115–25. doi: 10.1158/1078-0432.CCR-25-4896 (PMC13430218; doi:10.1158/1078-0432.CCR-25-4896)
Supplement: Figure S2 — Waterfall plot of maximum tumor shrinkage in patients evaluated at doses in dose expansion. [file ccr-25-4896_figure_s2_suppfs2.docx]

Figure S2


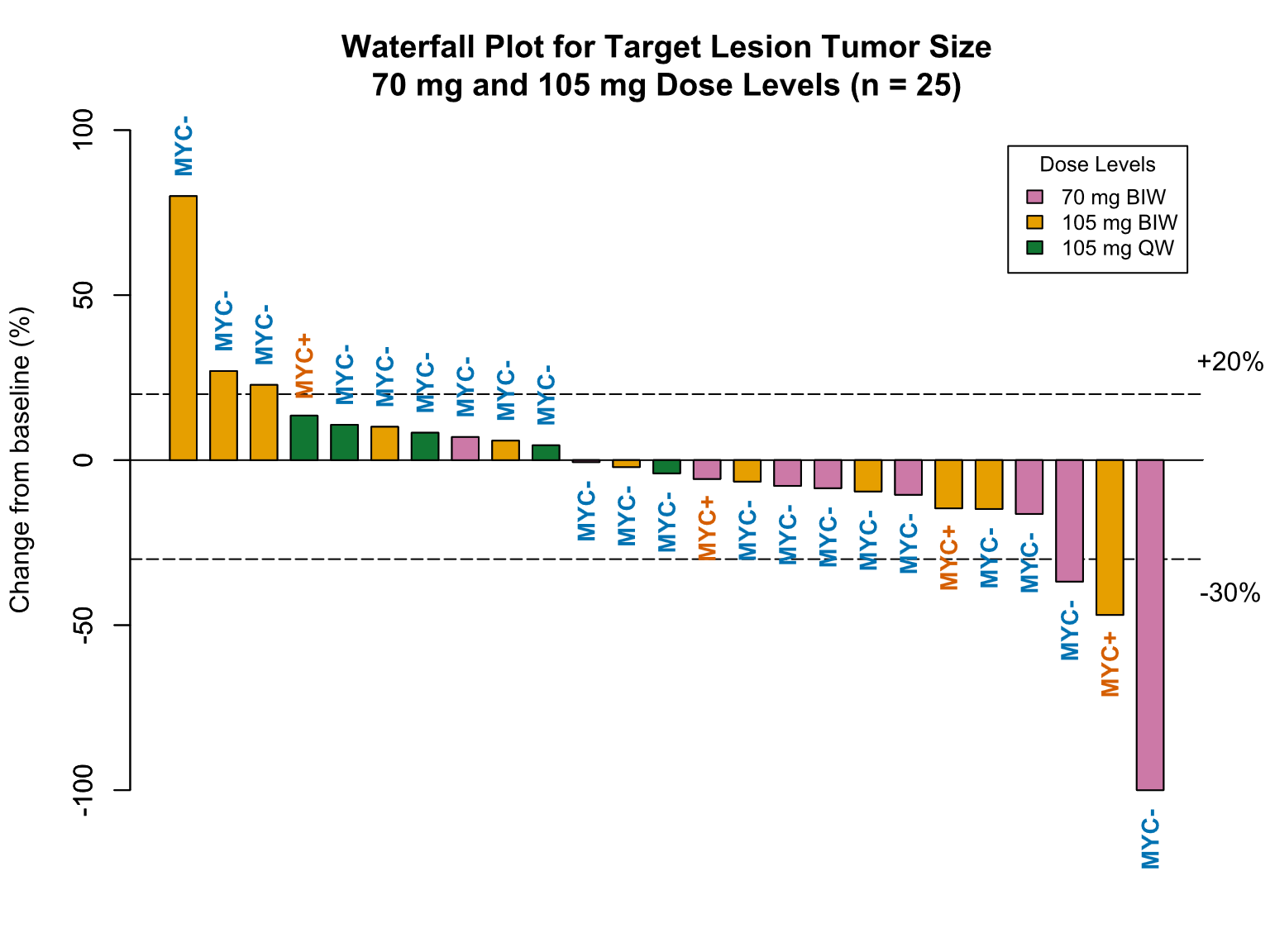


**Figure S3: Waterfall plot of maximum tumor shrinkage in patients evaluated at doses in dose expansion**

Changes in target lesion size from baseline are shown for patients treated with 70 mg BIW, 105 mg BIW, and 105 mg QW. The dashed lines indicate RECIST thresholds for tumor reduction or growth. MYC amplification status is indicated above or below each bar (amplification positive: MYC+; amplification negative: MYC-).
