# Supplementary material for: Phase I Study of Rogocekib in Patients with Advanced, Relapsed, or Refractory Malignant Solid Tumors
Source: Clin Cancer Res. 2026 May 18;32(15):3115–25. doi: 10.1158/1078-0432.CCR-25-4896 (PMC13430218; doi:10.1158/1078-0432.CCR-25-4896)
Supplement: Figure S3 — Waterfall plot of maximum tumor shrinkage in patients with ovarian cancer. [file ccr-25-4896_figure_s3_suppfs3.docx]

Figure S3


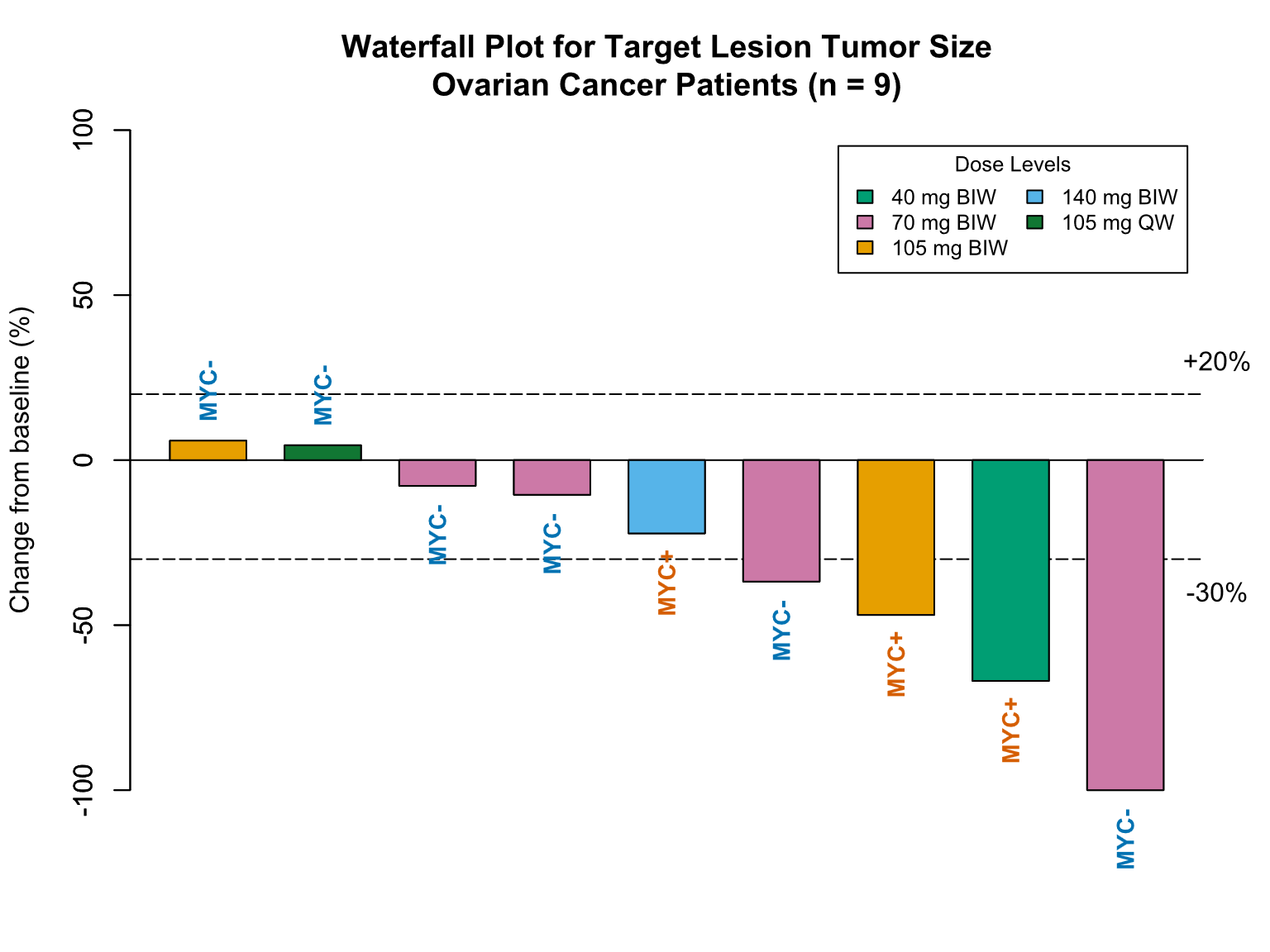


**Figure S3: Waterfall plot of maximum tumor shrinkage in patients with ovarian cancer.**

Changes in target lesion size from baseline are shown for patients with ovarian cancer and who had data available for MYC amplification status. The dashed lines indicate RECIST thresholds for tumor reduction or growth. MYC amplification status is indicated above or below each bar (amplification positive: MYC+; amplification negative: MYC-).
