# Supplementary material for: Phase I Study of Rogocekib in Patients with Advanced, Relapsed, or Refractory Malignant Solid Tumors
Source: Clin Cancer Res. 2026 May 18;32(15):3115–25. doi: 10.1158/1078-0432.CCR-25-4896 (PMC13430218; doi:10.1158/1078-0432.CCR-25-4896)
Supplement: Figure S4 — OS and PFS of all patients with solid tumors. [file ccr-25-4896_figure_s4_suppfs4.docx]

Figure S4


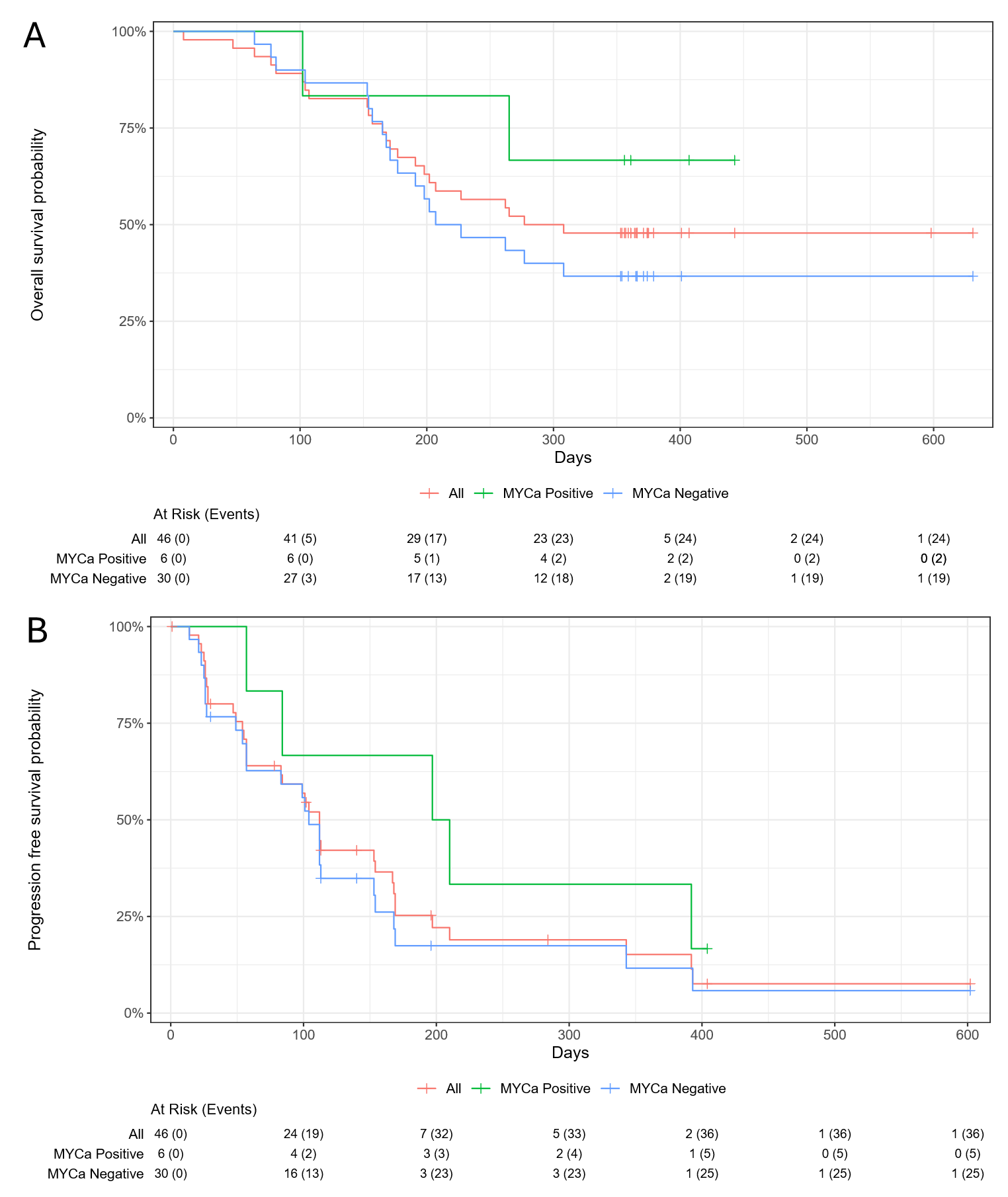


**Figure S4: OS and PFS of all patients with solid tumors**

Kaplan-Meier plots for all patients in CTX-712-Cl-01. Across all patients with solid tumors that were in the efficacy analysis population, the median OS (A) was 9.61 months (95% CI: 6.28 months-NR) while the median PFS (B) was 3.68 months (95% CI: 1.87-5.49 months) (A and B). The median OS was NR in all patients with MYC amplification.
