# Supplementary material for: Phase I Study of Rogocekib in Patients with Advanced, Relapsed, or Refractory Malignant Solid Tumors
Source: Clin Cancer Res. 2026 May 18;32(15):3115–25. doi: 10.1158/1078-0432.CCR-25-4896 (PMC13430218; doi:10.1158/1078-0432.CCR-25-4896)
Supplement: Figure S5 — Spider plot of changes in tumor size for all patients with ovarian cancer. [file ccr-25-4896_figure_s5_suppfs5.docx]

Figure S5


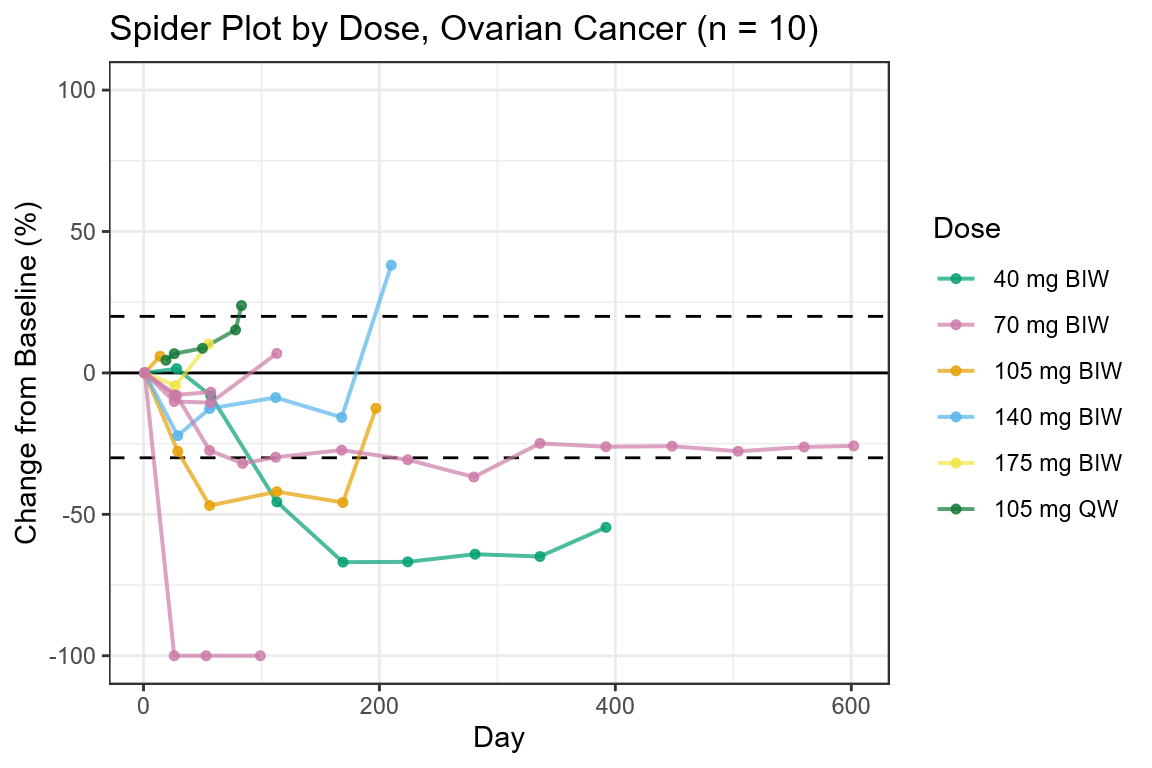


**Figure S5: Spider plot of changes in tumor size for all patients with ovarian cancer**

Spider plot of change in tumor size % over time were plotted for patients with ovarian cancer and separated by dose. At 10 mg and 20 mg dose levels, there were no patients with ovarian cancer. Only tumors with target lesions were graphed on this spider plot. Dashed lines indicate RECIST thresholds for tumor growth or reduction
