# Supplementary material for: Phase I Study of Rogocekib in Patients with Advanced, Relapsed, or Refractory Malignant Solid Tumors
Source: Clin Cancer Res. 2026 May 18;32(15):3115–25. doi: 10.1158/1078-0432.CCR-25-4896 (PMC13430218; doi:10.1158/1078-0432.CCR-25-4896)
Supplement: Figure S6 — OS and PFS of patients with ovarian cancer. [file ccr-25-4896_figure_s6_suppfs6.docx]

Figure S6


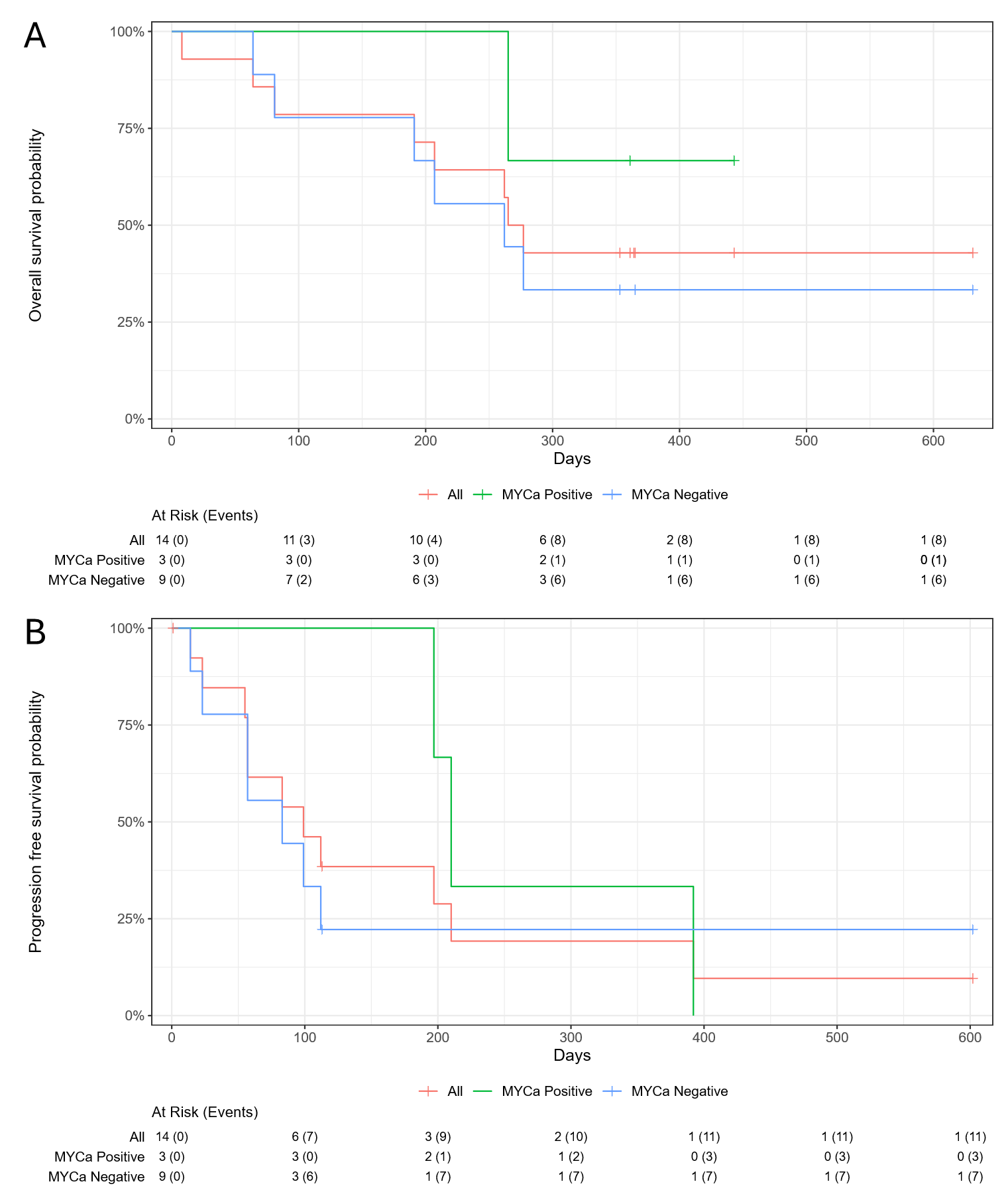


**Figure S6: OS and PFS of patients with ovarian cancer**

The median OS (A) in patients with ovarian cancer was 8.90 months (95% CI: 2.66 months-NR) and the PFS (B) was 3.25 months (95% CI: 1.81-6.90 months). Median OS NR in patients with ovarian cancer and MYC amplification.
