# Supplementary material for: Phase I Study of Rogocekib in Patients with Advanced, Relapsed, or Refractory Malignant Solid Tumors
Source: Clin Cancer Res. 2026 May 18;32(15):3115–25. doi: 10.1158/1078-0432.CCR-25-4896 (PMC13430218; doi:10.1158/1078-0432.CCR-25-4896)
Supplement: Figure S7 — OS and PFS for patients evaluated at doses in dose expansion. [file ccr-25-4896_figure_s7_suppfs7.docx]

Figure S7


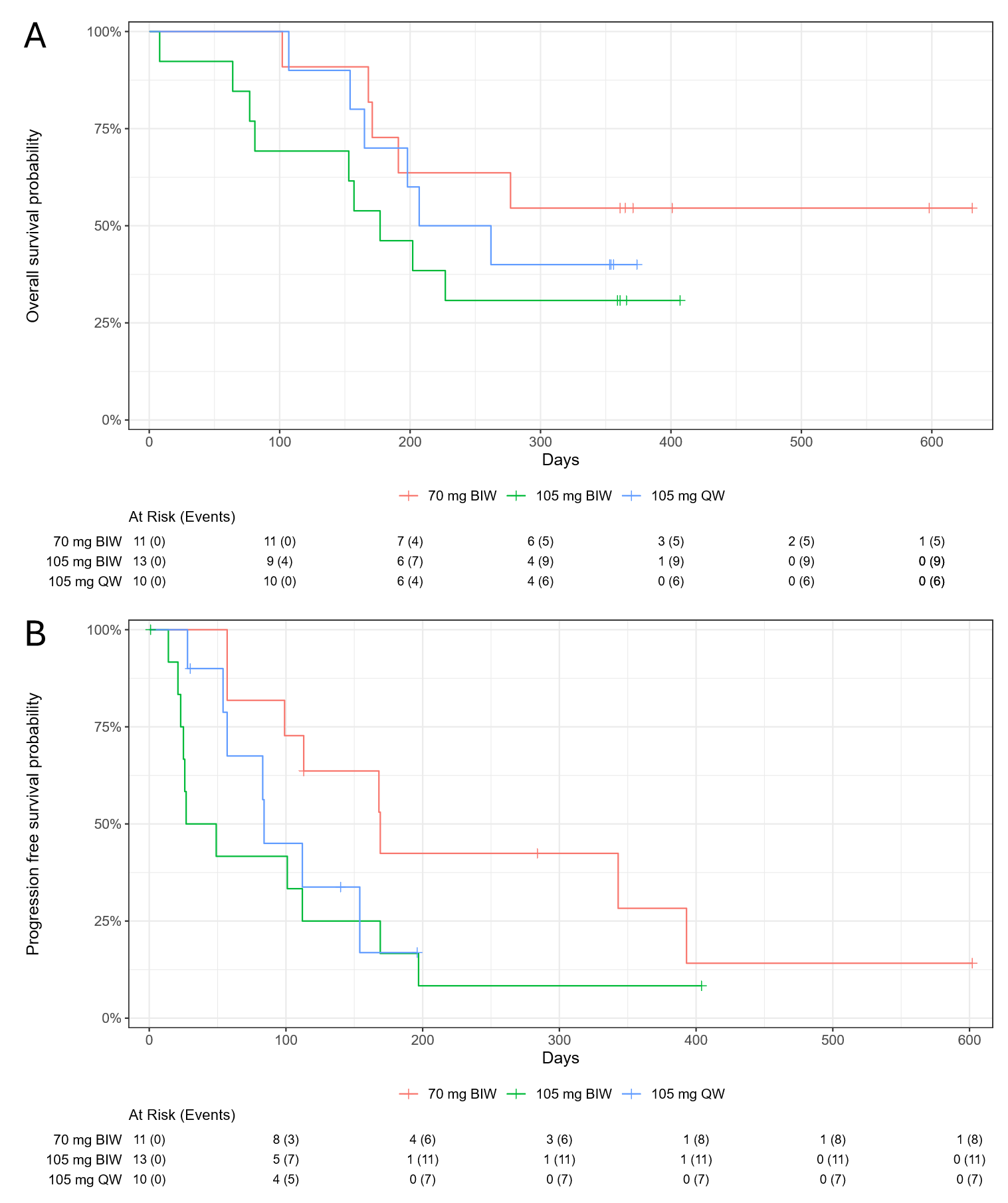


**Figure S7: OS and PFS for patients evaluated at doses in dose expansion**

Kaplan-Meier curves for both OS (A) and PFS (B) for patients who received doses that were evaluated during dose expansion. The median OS was not reached for the 70 mg TW group (95% CI: 6.28 months-NR), 5.82 months for the 105 mg TW group (95% CI: 2.66 months-NR), and 7.70 months for the 105 mg QW group (95% CI: 5.42 months-NR). The median PFS was 5.55 months for the 70 mg TW group (95% CI: 3.71 months-NR), 1.25 months for the 105 mg TW group (95% CI: 0.82 months-NR), and 2.76 months for the 105 mg QW group (95% CI: 1.87 months-NR).
