# Supplementary material for: Phase I Study of Rogocekib in Patients with Advanced, Relapsed, or Refractory Malignant Solid Tumors
Source: Clin Cancer Res. 2026 May 18;32(15):3115–25. doi: 10.1158/1078-0432.CCR-25-4896 (PMC13430218; doi:10.1158/1078-0432.CCR-25-4896)
Supplement: Table S1 — Primer and probe sequences for PD analysis. [file ccr-25-4896_table_s1_suppts1.docx]

Table S1: Primer and probe sequences for PD analysis

| **No.** | **Target gene** | **Type** | **Target RNA type** | **Sequence** |
| --- | --- | --- | --- | --- |
| 1 | *S6K* | Probe | non-skipping | 5’-FAM-TGGAAGACACTGCCTGCTTTTACTTGGC-MGB-3’ |
| 2 | *S6K* | Forward primer | non-skipping | 5’-TTATGCAGTTAGAAAGAGAGGGAATATTT-3’ |
| 3 | *S6K* | Reverse primer | non-skipping | 5’-CCCCAAAGCCATGGAGATTT-3’ |
| 4 | *S6K* | Probe | skipping | 5’-FAM-TGGAAGACACTGCCTGGTCATGTGAAA-MGB-3’ |
| 5 | *S6K* | Forward primer | skipping | 5’-TTATGCAGTTAGAAAGAGAGGGAATATTT-3’ |
| 6 | *S6K* | Reverse primer | skipping | 5’-GATTCTTTGCATAGTCCAAAGTCTGT-3’ |
| 7 | *THAP9-AS1* | Probe | non-skipping | 5’-VIC-TTGAAGGGTGTTTTTCT-MGB-3’ |
| 8 | *THAP9-AS1* | Forward primer | non-skipping | 5’-TTACTCAAAATATGCAGGGAAGGAA-3’ |
| 9 | *THAP9-AS1* | Reverse primer | non-skipping | 5’-GATCAAATAAATATGACAAATGATGTTCTCT-3’ |
| 10 | *THAP9-AS1* | Probe | skipping | 5’-FAM-CAATGAAGGAAATAAGGATCT-MGB-3’ |
| 11 | *THAP9-AS1* | Forward primer | skipping | 5’-ACCCTTTTCTGAAACACTTTGCC-3’ |
| 12 | *THAP9-AS1* | Reverse primer | skipping | 5’-TGATGTCTTATTCTTCTTAGTGCTTCAAAC-3’ |
