# Supplementary material for: Phase I Study of Rogocekib in Patients with Advanced, Relapsed, or Refractory Malignant Solid Tumors
Source: Clin Cancer Res. 2026 May 18;32(15):3115–25. doi: 10.1158/1078-0432.CCR-25-4896 (PMC13430218; doi:10.1158/1078-0432.CCR-25-4896)
Supplement: Table S2 — Representativeness of Study Participants. [file ccr-25-4896_table_s2_suppts2.docx]

Table S2: Representativeness of Study Participants

| Cancer types | Relapsed and Refractory Solid Tumors |
| --- | --- |
| Sex | According to the WHO Global Cancer Observatory, there were nearly 20 million new cases of cancer worldwide in 2022 with solid tumors making up more than 90% of all new cases. Of these, males represented 51.6% of all new cases (approximately 10.3 million cases) whereas females made up 48.4% of all new cases (approximately 9.7 million cases) (1). In Japan, there were nearly 1,000,000 new cases of cancer diagnosed in 2020 with males accounting for 56.6% of new cases (approximately 530,000 cases) and females accounting for 43.4% of new cases (approximately 410,000 cases) (2). |
| Age | In Japan, while cancer incidence is dependent on cancer type, overall cancer incidence showed a significant increase in people in their 60s and peaked for people in their 70s. The highest incidence of cancer occurred in patients aged 70-74 (2). |
| Race/ethnicity | In Japan, race/ethnicity data are not collected in Japan’s national cancer registry. Therefore, no statistics based on race or ethnicity are available for Japan. |
| Geography | Since 1981, cancer has been the leading cause of death in Japan (3) |
| Overall representativeness of this study | Even though this trial enrolled males and females that were close to balanced and in line with global statistics, there were more females in this trial than men (58.7% females vs 41.3% males). However, it is important to note that in this trial, inclusion criteria for cancer was narrowed in the expansion cohorts and in total, ovarian cancer accounted for approximately 30% of all patients enrolled which could explain the higher enrollment of females over males.  The median age of participants in this study was 56.0 years (range: 30-77 years) which is largely consistent with the median age of patients enrolled in trials across Japan (4). While this study is not entirely representative of cancer patients within Japan, there exist barriers to enrolling elderly patients in trials within Japan such as trial design (restrictive inclusion criteria) or physicians tending to have a negative bias towards enrolling elderly patients.  This trial was conducted at one site in Tokyo, Japan. The National Cancer Center Hospital functions as a major national referral center for cancer care in Japan. Because of this, patients across the country travel from both rural and urban areas to seek treatment, although most patients come from urban areas. However, for this early-phase clinical trial, the utilization of a single site does not significantly impact the objectives of this trial.  Additionally, 100% of the trial participants were Asian which largely aligns with the homogenous overall population of Japan. While this study does not fully represent the global population of cancer patients, further studies are being planned for the future which include sites like the US where a more diverse patient population can be investigated. |

1. Bray F, Laversanne M, Sung H, Ferlay J, Siegel RL, Soerjomataram I, et al. Global cancer statistics 2022: GLOBOCAN estimates of incidence and mortality worldwide for 36 cancers in 185 countries. CA: A Cancer Journal for Clinicians. 2024;74:229–63.

2. CANCER STATISTICS IN JAPAN 2025 [Internet]. Available from: https://ganjoho.jp/public/qa_links/report/statistics/2025_en.html

3. Matsuda T, Saika K. Cancer burden in Japan based on the latest cancer statistics: need for evidence-based cancer control programs. Annals of Cancer Epidemiology. AME Publishing Company; 2018;2.

4. Yonemori K, Hirakawa A, Komiyama N, Kouno T, Ando M, Fujiwara Y, et al. Participation of elderly patients in registration trials for oncology drug applications in Japan†. Annals of Oncology. Elsevier; 2010;21:2112–8.
