# Supplementary material for: Phase I Study of Rogocekib in Patients with Advanced, Relapsed, or Refractory Malignant Solid Tumors
Source: Clin Cancer Res. 2026 May 18;32(15):3115–25. doi: 10.1158/1078-0432.CCR-25-4896 (PMC13430218; doi:10.1158/1078-0432.CCR-25-4896)
Supplement: Table S3 — Expanded previous therapies for patients with solid tumors. [file ccr-25-4896_table_s3_suppts3.docx]

Table S3: Expanded previous therapies for patients with solid tumors

|  | Cohort A:  10 mg | | Cohort A:  20 mg | | Cohort A:  40 mg | | Cohort A:  70 mg | | Cohort A:  105 mg | | Cohort A:  140 mg | | Cohort A:  175 mg | | Cohort C:  105mg^*1^ | | Cohort D:  70mg | | Cohort E:  105mg^*2^ | |
| --- | --- | --- | --- | --- | --- | --- | --- | --- | --- | --- | --- | --- | --- | --- | --- | --- | --- | --- | --- | --- |
|  | n=1 | | n=1 | | n=1 | | n=1 | | n=3 | | n=6 | | n=3 | | n=10 | | n=10 | | n=10 | |
|  | n (%) | | n (%) | | n (%) | | n (%) | | n (%) | | n (%) | | n (%) | | n (%) | | n (%) | | n (%) | |
| Prior Systemic Therapy | | | | | | | | | | | | | | | | | | | | |
| No | 0 | (0.0) | 0 | (0.0) | 0 | (0.0) | 0 | (0.0) | 0 | (0.0) | 0 | (0.0) | 0 | (0.0) | 0 | (0.0) | 0 | (0.0) | 0 | (0.0) |
| Yes | 1 | (100.0) | 1 | (100.0) | 1 | (100.0) | 1 | (100.0) | 3 | (100.0) | 6 | (100.0) | 3 | (100.0) | 10 | (100.0) | 10 | (100.0) | 10 | (100.0) |
| Reason for Prior Systemic Therapy | | | | | | | | | | | | | | | | | | | | |
| Adjuvant | 0 | | 0 | | 1 | | 0 | | 1 | | 1 | | 0 | | 6 | | 3 | | 4 | |
| Advanced or Recurrent | 1 | | 1 | | 1 | | 1 | | 3 | | 6 | | 3 | | 10 | | 10 | | 10 | |
| Other | 1 | | 0 | | 0 | | 0 | | 1 | | 0 | | 1 | | 1 | | 2 | | 1 | |
| Prior Radiation Therapy | | | | | | | | | | | | | | | | | | | | |
| No | 0 | (0.0) | 1 | (100.0) | 1 | (100.0) | 1 | (100.0) | 1 | (33.3) | 3 | (50.0) | 1 | (33.3) | 8 | (80.0) | 9 | (90.0) | 3 | (30.0) |
| Yes | 1 | (100.0) | 0 | (0.0) | 0 | (0.0) | 0 | (0.0) | 2 | (66.7) | 3 | (50.0) | 2 | (66.7) | 2 | (20.0) | 1 | (10.0) | 7 | (70.0) |
| Procedure/Surgery/Stem Cell Transplant History | | | | | | | | | | | | | | | | | | | | |
| No | 0 | (0.0) | 0 | (0.0) | 0 | (0.0) | 0 | (0.0) | 0 | (0.0) | 3 | (50.0) | 1 | (33.3) | 1 | (10.0) | 2 | (20.0) | 0 | (0.0) |
| Yes | 1 | (100.0) | 1 | (100.0) | 1 | (100.0) | 1 | (100.0) | 3 | (100.0) | 3 | (50.0) | 2 | (66.7) | 9 | (90.0) | 8 | (80.0) | 10 | (100.0) |

*1: 105mg Twice a week

*2: 105mg Once a week
