# Supplementary material for: Phase I Study of Rogocekib in Patients with Advanced, Relapsed, or Refractory Malignant Solid Tumors
Source: Clin Cancer Res. 2026 May 18;32(15):3115–25. doi: 10.1158/1078-0432.CCR-25-4896 (PMC13430218; doi:10.1158/1078-0432.CCR-25-4896)
Supplement: Table S4 — All TRAEs occurring in ≥10% of patients with solid tumors. [file ccr-25-4896_table_s4_suppts4.docx]

Table S4: All TRAEs occurring in ≥10% of patients with solid tumors

|  | | Solid Tumor Cohort (n=46) | | | | | | | | | | | |
| --- | --- | --- | --- | --- | --- | --- | --- | --- | --- | --- | --- | --- | --- |
|  |  | Treatment Related Adverse Events | | | | | | | | | | | |
|  |  | Grade 1 | | Grade 2 | | Grade 3 | | Grade 4 | | Grade 5 | | All Grade | |
|  |  | n (%)^*1^ | | n (%)^*1^ | | n (%)^*1^ | | n (%)^*1^ | | n (%)^*1^ | | n (%)^*1^ | |
| Any Events | | 10 | (21.7) | 29 | (63.0) | 4 | (8.7) | 1 | (2.2) | 2 | (4.3) | 46 | (100.0) |
|  | Nausea | 28 | (60.9) | 15 | (32.6) | 0 | (0.0) | 0 | (0.0) | 0 | (0.0) | 43 | (93.5) |
|  | Vomiting | 21 | (45.7) | 9 | (19.6) | 0 | (0.0) | 0 | (0.0) | 0 | (0.0) | 30 | (65.2) |
|  | Diarrhoea | 17 | (37.0) | 7 | (15.2) | 1 | (2.2) | 0 | (0.0) | 0 | (0.0) | 25 | (54.3) |
|  | Decreased appetite | 9 | (19.6) | 12 | (26.1) | 0 | (0.0) | 0 | (0.0) | 0 | (0.0) | 21 | (45.7) |
|  | Blood creatinine increased | 13 | (28.3) | 2 | (4.3) | 0 | (0.0) | 0 | (0.0) | 0 | (0.0) | 15 | (32.6) |
|  | Malaise | 8 | (17.4) | 2 | (4.3) | 0 | (0.0) | 0 | (0.0) | 0 | (0.0) | 10 | (21.7) |
|  | Weight decreased | 5 | (10.9) | 5 | (10.9) | 0 | (0.0) | 0 | (0.0) | 0 | (0.0) | 10 | (21.7) |
|  | Dysgeusia | 9 | (19.6) | 1 | (2.2) | 0 | (0.0) | 0 | (0.0) | 0 | (0.0) | 10 | (21.7) |
|  | Aspartate aminotransferase increased | 7 | (15.2) | 2 | (4.3) | 0 | (0.0) | 0 | (0.0) | 0 | (0.0) | 9 | (19.6) |
|  | White blood cell count decreased | 6 | (13.0) | 3 | (6.5) | 0 | (0.0) | 0 | (0.0) | 0 | (0.0) | 9 | (19.6) |
|  | Anaemia | 3 | (6.5) | 5 | (10.9) | 0 | (0.0) | 0 | (0.0) | 0 | (0.0) | 8 | (17.4) |
|  | Alanine aminotransferase increased | 6 | (13.0) | 2 | (4.3) | 0 | (0.0) | 0 | (0.0) | 0 | (0.0) | 8 | (17.4) |
|  | Platelet count decreased | 4 | (8.7) | 0 | (0.0) | 3 | (6.5) | 0 | (0.0) | 0 | (0.0) | 7 | (15.2) |
|  | Rash | 6 | (13.0) | 1 | (2.2) | 0 | (0.0) | 0 | (0.0) | 0 | (0.0) | 7 | (15.2) |
|  | Fatigue | 3 | (6.5) | 3 | (6.5) | 0 | (0.0) | 0 | (0.0) | 0 | (0.0) | 6 | (13.0) |
|  | Pyrexia | 4 | (8.7) | 2 | (4.3) | 0 | (0.0) | 0 | (0.0) | 0 | (0.0) | 6 | (13.0) |
|  | Dehydration | 0 | (0.0) | 5 | (10.9) | 1 | (2.2) | 0 | (0.0) | 0 | (0.0) | 6 | (13.0) |
|  | Headache | 6 | (13.0) | 0 | (0.0) | 0 | (0.0) | 0 | (0.0) | 0 | (0.0) | 6 | (13.0) |
|  | Constipation | 5 | (10.9) | 0 | (0.0) | 0 | (0.0) | 0 | (0.0) | 0 | (0.0) | 5 | (10.9) |

*1: If a patient is reported to have the same event more than once, then the event with the most severe grade is counted once.
