# Supplementary material for: Phase I Study of Rogocekib in Patients with Advanced, Relapsed, or Refractory Malignant Solid Tumors
Source: Clin Cancer Res. 2026 May 18;32(15):3115–25. doi: 10.1158/1078-0432.CCR-25-4896 (PMC13430218; doi:10.1158/1078-0432.CCR-25-4896)
Supplement: Table S5 — Dose intensity per dose level in patients with solid tumors. [file ccr-25-4896_table_s5_suppts5.docx]

Table S5: Dose intensity per dose level in patients with solid tumors

|  | Cohort A:  10 mg | Cohort A:  20 mg | Cohort A:  40 mg | Cohort A:  70 mg | Cohort A:  105 mg | Cohort A:  140 mg | Cohort A:  175 mg | Cohort C:  105mg^*1^ | Cohort D:  70mg | Cohort E:  105mg^*2^ |
| --- | --- | --- | --- | --- | --- | --- | --- | --- | --- | --- |
|  | n=1 | n=1 | n=1 | n=1 | n=3 | n=6 | n=3 | n=10 | n=10 | n=10 |
|  | n (%) | n (%) | n (%) | n (%) | n (%) | n (%) | n (%) | n (%) | n (%) | n (%) |
| Actual Dose Intensity (mg/week) | | | | | | | | | | |
| Mean (S.D.) | 19.33 (-) | 40.00 (-) | 57.14 (-) | 90.00 (-) | 164.19 (36.82) | 227.25 (40.79) | 246.81 (64.54) | 170.38 (49.76) | 105.16 (34.13) | 97.65 (11.20) |
| Median | 19.33 | 40.00 | 57.14 | 90.00 | 161.00 | 217.29 | 265.42 | 196.88 | 109.38 | 105.00 |
| IQR [Q1, Q3] | [19.3, 19.3] | [40.0, 40.0] | [57.1, 57.1] | [90.0, 90.0] | [129.1, 202.5] | [193.3, 270.7] | [175.0, 300.0] | [105.0, 210.0] | [82.2, 140.0] | [91.9, 105.0] |
| Range [Min, Max] | [19.3, 19.3] | [40.0, 40.0] | [57.1, 57.1] | [90.0, 90.0] | [129.1, 202.5] | [185.0, 280.0] | [175.0, 300.0] | [95.0, 210.0] | [45.4, 140.0] | [76.4, 105.0] |
| Relative Dose Intensity (%) | | | | | | | | | | |
| Mean (S.D.) | 96.67 (-) | 100.00 (-) | 71.43 (-) | 64.29 (-) | 78.18 (17.53) | 81.16 (14.57) | 70.52 (18.44) | 81.13 (23.70) | 75.12 (24.38) | 93.00 (10.67) |
| Median | 96.67 | 100.00 | 71.43 | 64.29 | 76.67 | 77.60 | 75.83 | 93.75 | 78.13 | 100.00 |
| IQR [Q1, Q3] | [96.7, 96.7] | [100.0, 100.0] | [71.4, 71.4] | [64.3, 64.3] | [61.5, 96.4] | [69.0, 96.7] | [50.0, 85.7] | [50.0, 100.0] | [58.7, 100.0] | [87.5, 100.0] |
| Range [Min, Max] | [96.7, 96.7] | [100.0, 100.0] | [71.4, 71.4] | [64.3, 64.3] | [61.5, 96.4] | [66.1, 100.0] | [50.0, 85.7] | [45.2, 100.0] | [32.4, 100.0] | [72.7, 100.0] |
| Relative Dose Intensity Category (%) | | | | | | | | | | |
| < 80% | 0 (0.0) | 0 (0.0) | 1 (100.0) | 1 (100.0) | 2 (66.7) | 3 (50.0) | 2 (66.7) | 4 (40.0) | 5 (50.0) | 2 (20.0) |
| 80% ≤ < 90% | 0 (0.0) | 0 (0.0) | 0 (0.0) | 0 (0.0) | 0 (0.0) | 1 (16.7) | 1 (33.3) | 1 (10.0) | 1 (10.0) | 1 (10.0) |
| 90% ≤ | 1 (100.0) | 1 (100.0) | 0 (0.0) | 0 (0.0) | 1 (33.3) | 2 (33.3) | 0 (0.0) | 5 (50.0) | 4 (40.0) | 7 (70.0) |

*1: 105mg Twice a week

*2: 105mg Once a week
