# Supplementary material for: Phase I Study of Rogocekib in Patients with Advanced, Relapsed, or Refractory Malignant Solid Tumors
Source: Clin Cancer Res. 2026 May 18;32(15):3115–25. doi: 10.1158/1078-0432.CCR-25-4896 (PMC13430218; doi:10.1158/1078-0432.CCR-25-4896)
Supplement: Table S6 — Biomarker analysis of tissue samples from patients with solid tumors. [file ccr-25-4896_table_s6_suppts6.docx]

Table S6: Biomarker analysis of tissue samples from patients with solid tumors

| **Analyzed samples** | | **CNV** | **Short Variant** | |
| --- | --- | --- | --- | --- |
| **ID** | **Visit Code** | ***MYC*** | ***SF3B1*** | ***U2AF1*** |
| Patient 03 | SCR | GAIN |  |  |
| Patient 05 | SCR | GAIN |  |  |
| Patient 06 | SCR |  | p.K700E |  |
| Patient 07 | SCR |  |  |  |
| Patient 08 | SCR | GAIN |  |  |
| Patient 09 | SCR |  |  |  |
| Patient 10 | SCR |  |  |  |
| Patient 14 | SCR |  |  |  |
| Patient 16 | SCR |  |  |  |
| Patient 17 | SCR |  |  |  |
| Patient 17 | C3D1 |  |  |  |
| Patient 18 | SCR |  |  |  |
| Patient 19 | SCR |  |  |  |
| Patient 20 | SCR |  |  |  |
| Patient 22 | SCR |  |  |  |
| Patient 23 | SCR | GAIN |  |  |
| Patient 23 | C3D1 | GAIN |  |  |
| Patient 24 | SCR |  |  |  |
| Patient 25 | SCR |  |  |  |
| Patient 26 | SCR |  |  |  |
| Patient 27 | SCR |  |  |  |
| Patient 28 | SCR |  |  |  |
| Patient 29 | SCR | GAIN |  |  |
| Patient 30 | SCR |  |  |  |
| Patient 31 | SCR | GAIN |  |  |
| Patient 32 | SCR |  |  |  |
| Patient 33 | SCR |  |  |  |
| Patient 34 | SCR |  |  |  |
| Patient 36 | SCR |  |  |  |
| Patient 37 | SCR |  |  |  |
| Patient 38 | SCR |  |  |  |
| Patient 40 | SCR |  |  |  |
| Patient 41 | SCR |  |  |  |
| Patient 42 | SCR |  |  |  |
| Patient 43 | SCR |  |  |  |
| Patient 44 | SCR |  |  |  |
| Patient 45 | SCR |  |  |  |
| Patient 46 | SCR |  |  |  |

GAIN indicates copy number amplification.

SCR indicates that samples were either collected during screening or from previous archives.

C3D1 indicates that samples were collected around Cycle 3 day 1.

Note: Only 36 patients had tissue samples available for analysis.
