# Supplementary material for: Phase I Study of Rogocekib in Patients with Advanced, Relapsed, or Refractory Malignant Solid Tumors
Source: Clin Cancer Res. 2026 May 18;32(15):3115–25. doi: 10.1158/1078-0432.CCR-25-4896 (PMC13430218; doi:10.1158/1078-0432.CCR-25-4896)
Supplement: Table S7 — ORR of all patients with solid tumors and ovarian cancer. [file ccr-25-4896_table_s7_suppts7.docx]

Table S7: ORR of all patients with solid tumors and ovarian cancer

|  | All Solid Tumors | | Ovarian Cancer | |
| --- | --- | --- | --- | --- |
|  | N=46 | | N=14 | |
|  | N(%) | 95% CI^*1^ | N(%) | 95% CI^*1^ |
| Best Overall Response | | | | |
| CR | 0 (0.0) | [0.0-7.7] | 0 (0.0) | [0.0-23.2] |
| PR | 3 (6.5) | [1.4-17.9] | 3 (21.4) | [4.7-50.8] |
| SD | 21 (45.7) | [30.9-61.0] | 4 (28.6) | [8.4-58.1] |
| Non-CR/Non-PD | 3 (6.5) | [1.4-17.9] | 1 (7.1) | [0.2-33.9] |
| PD | 16 (34.8) | [21.4-50.2] | 5 (35.7) | [12.8-64.9] |
| NE | 3 (6.5) | [1.4-17.9] | 1 (7.1) | [0.2-33.9] |
| ORR | 3 (6.5) | [1.4-17.9] | 3 (21.4) | [4.7-50.8] |

*1 Clopper-Pearson exact method
